# Supplementary material for: Reproductive seasonality in the Baka Pygmies, environmental factors and climatic changes
Source: PLoS One. 2022 Mar 8;17(3):e0264761. doi: 10.1371/journal.pone.0264761 (PMC8903253; doi:10.1371/journal.pone.0264761)
Supplement: S1 Text — (PDF) [file pone.0264761.s001.pdf]

Seasonality of birth in the Baka Pygmies, environmental factors and climatic changes

Laura Piqué-Fandiño[1], Sandrine Gallois[2], Samuel Pavard[1¶] and Fernando V. Ramirez Rozzi[1,3 ¶*]

[1] Eco-anthropologie (EA), Muséum national d'Histoire naturelle, CNRS, Université de Paris, Musée de l'Homme Paris, France.

[2] Universitat oberta de Catalunya, Barcelona, Spain.

[3] EA 2496, UP, Faculté de Chirurgie Dentaire, Montrouge, France

¶ These authors supervised this study equally

Corresponding author: Fernando V. Ramirez Rozzi, fernando.ramirez-rozzi@mnhn.fr

Keywords: Hunter-gatherer, human fertility, mobility, socio-cultural practices, birth pattern

Supplementary Information

Supplementary Text

Number of births is a valid method to assess seasonality in births

To check whether the monthly number of births provides valid data to test for seasonality, we used two sets of data: first, the monthly number of births from 2007 to 2018; secondly, the monthly fertility rates (the number of births per month divided by the number of women giving birth per year) (S3 Table). On the one hand, we wanted to keep the inter-annual variance in order to account for temporal fluctuations in births from year to year, but on the other hand, the small sample size means large fluctuations in the observed number of births for any given month in a given year. To keep the variance while decreasing yearly and monthly variance, we chose to smooth both sets of data across three successive years and three successive months using the 'smooth' function in 'R' (ref R, kind "3", end rule "copy").

We calculated the absolute and relative deviation from the yearly average smoothed fertility rates and number of births, and used these to build boxplots (Supplementary Figure 2) showing the raw data distribution. We then generated locally weighted polynomial fits (Supplementary Figure 1) using the 'locally.weighted.polynomial' of the SiZer library in 'R' to provide smoothed data.

Additionally, we correlated fertility rates (S3 Table) with both the number of births and the monthly deviation from the averaged number of births.

The fluctuation in the fertility rate over the year (S1 Fig A) for the period 2007-2018 is similar to that in the monthly deviations from the annual average number of births (S1 Fig B). The correlations between fertility rates and number of births are significant (correlation factor=0.9389, p<0.001), as well as between fertility rates and monthly deviations from the mean number of births (correlation factor=0.9022, p<0.001). The correlation between the two metrics (i.e., deviation in number of birth and in fertility rates) is about 94% and fluctuations over years show no dissimilarities (S2 Fig). The monthly number of births constitutes a valid method to determine birth seasonality in this sample and the analyses can be extended to years before 2007, for which we do not have data on women present in the village at the time or on fertility rates.
