# Supplementary material for: Reproductive seasonality in the Baka Pygmies, environmental factors and climatic changes
Source: PLoS One. 2022 Mar 8;17(3):e0264761. doi: 10.1371/journal.pone.0264761 (PMC8903253; doi:10.1371/journal.pone.0264761)
Supplement: S1 Table — (PDF) [file pone.0264761.s002.pdf]

S1 Table. Precipitation and average daily precipitation by month and year (mm).

Year	Jan	Feb	Mar	Apr	May	Jun	Jul	Aug	Sep	Oct	Nov	Dec	X day/yr
1980	2.018	2.398	5.890	7.042	3.681	5.337	5.497	6.008	4.440	5.934	5.703	1.619	4.631
1981	1.841	2.022	4.383	5.508	5.867	3.693	5.011	5.531	6.660	5.405	5.070	2.579	4.464
1982	4.416	2.107	4.852	3.994	4.166	4.883	4.189	5.390	7.729	6.296	3.635	3.008	4.555
1988	1.932	3.542	5.833	5.730	5.356	3.563	3.857	4.650	6.062	6.948	6.525	3.304	4.775
1989	0.280	0.294	4.034	4.730	5.440	3.006	3.544	6.142	5.447	5.875	5.835	2.426	3.921
1990	1.883	1.144	3.540	4.637	4.349	2.403	3.948	5.512	5.054	6.695	5.497	3.225	3.991
1991	1.041	1.562	2.834	5.169	4.982	3.353	4.318	4.875	5.545	5.074	5.787	0.669	3.767
1992	0.631	0.439	4.827	4.744	4.723	4.639	5.650	4.799	5.173	6.084	4.589	1.688	3.999
1993	1.831	1.530	4.368	5.774	4.021	4.807	4.322	7.868	5.520	5.783	7.364	2.750	4.661
1994	2.743	1.116	5.018	5.581	4.421	5.836	6.041	7.381	8.377	8.196	5.283	0.338	5.028
1995	0.593	1.785	5.878	5.493	5.535	5.085	6.432	7.484	6.332	8.059	3.201	1.646	4.794
1996	0.980	2.121	7.099	5.486	5.562	4.482	3.624	5.394	4.936	8.286	2.338	1.856	4.347
1997	1.963	0.099	4.383	4.509	3.811	3.738	3.647	4.936	5.260	7.694	5.589	1.591	3.935
1998	0.690	0.809	2.743	5.001	3.555	3.551	4.704	5.821	5.810	6.119	5.411	2.579	3.899
1999	1.413	2.527	4.158	7.725	4.597	4.097	5.566	8.461	6.760	7.278	4.417	0.904	4.825
2000	0.902	1.108	2.989	5.804	5.116	5.047	3.349	4.665	7.980	6.844	4.320	1.064	4.099
2001	0.401	0.498	6.565	6.371	3.765	3.910	3.922	3.334	5.219	6.775	4.135	2.228	3.927
2002	0.532	1.965	4.044	4.822	3.681	3.222	3.586	4.524	4.734	6.348	5.581	1.631	3.722
2003	1.686	1.551	4.539	5.463	4.711	4.738	3.216	4.288	5.154	4.463	3.380	1.144	3.694
2004	1.505	0.767	2.434	7.240	3.082	3.746	5.577	4.486	5.367	5.341	4.951	0.599	3.758
2005	0.111	1.015	5.409	2.781	3.372	2.541	2.092	3.479	5.602	6.290	2.495	2.302	3.124
2006	1.438	1.513	2.872	5.083	3.902	2.262	3.098	2.708	5.421	6.388	3.693	0.463	3.237
2007	0.114	1.146	2.472	5.646	5.802	2.693	3.180	6.222	5.951	7.042	3.124	0.883	3.690
2008	1.451	0.399	3.876	6.460	3.551	2.518	3.136	4.402	4.906	4.766	2.327	2.506	3.358
2009	0.814	3.201	3.428	4.215	4.597	3.674	2.796	4.604	4.816	5.362	4.326	1.003	3.570
2010	0.547	1.797	3.479	5.033	4.307	2.926	4.040	3.254	6.872	5.497	5.962	1.017	3.728
2011	0.290	2.611	1.904	3.370	3.637	2.390	2.714	4.532	4.684	6.887	4.894	0.284	3.183
2012	0.296	2.237	2.464	3.664	4.311	4.021	2.964	5.497	5.116	5.222	4.715	1.369	3.490
2013	1.205	1.621	4.711	3.334	4.215	2.537	3.769	3.819	5.791	5.116	4.175	1.795	3.507
2014	0.402	1.598	3.899	6.386	5.882	3.113	2.449	4.742	4.913	5.730	4.707	1.230	3.754
2015	0.479	2.234	3.998	4.013	3.330	3.349	2.689	4.173	4.066	6.855	4.263	0.263	3.309
2016	0.381	0.456	4.597	5.207	5.146	4.280	3.571	3.784	5.135	5.455	4.955	2.012	3.748
2017	0.891	0.599	2.361	6.170	3.613	4.005	3.880	4.108	5.699	5.320	4.238	0.668	3.463
2018	0.195	2.760	3.757	3.784	3.716	2.651	3.159	3.637	4.385	5.688	3.929	1.640	3.275
**X day/month**	1.115	1.546	4.107	5.176	4.406	3.709	3.927	5.015	5.615	6.209	4.600	1.597	**3.918**
