# Supplementary material for: Reproductive seasonality in the Baka Pygmies, environmental factors and climatic changes
Source: PLoS One. 2022 Mar 8;17(3):e0264761. doi: 10.1371/journal.pone.0264761 (PMC8903253; doi:10.1371/journal.pone.0264761)
Supplement: S2 Table — (PDF) [file pone.0264761.s003.pdf]

S2 Table. Temperature and average daily temperature by month and year (°C).

Year	Jan	Feb	Mar	Apr	May	Jun	Jul	Aug	Sep	Oct	Nov	Dec	X
1980	23.280	24.073	24.183	24.226	23.517	22.833	22.188	22.463	22.674	22.706	22.216	22.390	23.063
1981	22.804	24.038	23.820	23.711	23.120	23.071	22.278	22.585	22.441	22.630	22.509	22.741	22.980
1982	22.741	23.536	24.018	24.049	23.280	22.816	22.394	22.362	22.548	22.029	22.747	22.697	22.935
1988	23.446	24.432	24.245	24.391	23.840	23.064	22.510	23.029	22.738	22.573	22.900	22.495	23.306
1989	21.940	24.098	24.004	23.949	23.008	23.313	22.406	22.395	22.713	22.417	23.269	22.947	23.039
1990	23.185	24.293	25.017	24.565	23.751	23.723	22.589	22.734	22.810	22.875	23.218	23.398	23.514
1991	23.826	25.160	25.232	23.950	23.469	23.858	22.884	22.697	23.010	21.995	22.437	23.309	23.486
1992	23.746	24.699	24.766	24.001	23.408	22.829	22.224	22.270	22.355	22.337	22.686	22.991	23.193
1993	22.793	24.187	24.389	24.285	24.074	23.270	22.740	22.715	22.689	23.033	22.886	22.913	23.332
1994	23.310	24.331	24.800	24.351	23.753	23.189	22.733	22.858	22.944	22.362	22.867	23.801	23.442
1995	24.255	24.317	24.408	24.643	23.783	23.506	22.951	23.206	23.117	22.545	23.248	23.182	23.597
1996	24.120	24.388	23.956	24.022	23.424	23.127	22.640	22.871	22.967	22.316	23.213	23.012	23.338
1997	23.554	24.428	24.989	23.959	23.494	23.139	23.287	23.512	23.228	22.986	23.149	23.499	23.603
1998	24.021	26.497	26.387	25.461	24.976	24.185	23.343	23.240	23.044	22.860	23.177	23.355	24.213
1999	23.173	24.120	24.268	23.715	23.328	23.093	22.477	22.621	22.391	22.188	22.687	23.156	23.102
2000	23.551	23.968	24.733	23.795	23.638	22.819	22.443	22.608	22.385	22.596	22.792	22.862	23.183
2001	23.103	24.960	23.676	23.896	23.881	22.845	22.504	22.743	22.532	22.792	23.022	23.468	23.286
2002	23.356	24.592	24.334	24.216	24.229	23.388	23.304	22.981	23.025	22.511	22.902	23.532	23.531
2003	24.134	25.364	24.851	24.229	23.847	23.121	23.189	23.293	22.947	22.974	23.442	23.894	23.774
2004	24.269	24.754	25.241	24.416	23.560	22.781	22.697	23.139	22.889	22.730	23.211	23.956	23.637
2005	24.584	25.913	25.100	24.946	24.227	23.507	23.245	23.050	23.303	22.794	23.629	23.137	23.953
2006	24.107	24.681	24.322	24.112	23.258	23.490	23.332	23.382	22.773	23.074	23.103	23.566	23.601
2007	23.843	25.563	25.577	24.381	24.004	23.223	23.081	22.949	22.829	22.759	23.119	23.424	23.730
2008	23.684	24.533	24.095	23.820	23.711	23.474	23.005	23.268	23.183	23.140	23.622	23.379	23.576
2009	23.972	24.275	24.787	24.303	24.037	23.392	23.427	23.497	23.786	23.288	23.611	23.873	23.854
2010	24.579	25.537	25.357	25.371	24.812	24.015	23.282	23.476	23.255	23.180	23.309	23.472	24.138
2011	23.459	24.051	25.133	24.543	24.471	23.703	23.238	23.009	23.199	22.893	23.089	23.605	23.700
2012	24.511	24.559	25.213	24.635	23.808	23.507	23.126	23.182	23.057	23.173	23.561	23.543	23.823
2013	24.625	25.545	24.566	24.741	24.356	23.848	22.779	23.099	23.241	22.931	23.325	23.136	23.850
2014	24.555	25.048	24.944	24.205	24.009	23.809	23.404	23.070	23.227	23.403	23.657	23.641	23.915
2015	23.346	24.882	24.951	24.598	24.453	23.588	23.952	23.779	23.571	23.481	23.714	23.341	23.972
2016	24.516	26.479	25.604	25.341	24.292	23.696	23.548	23.564	23.684	23.636	23.711	23.718	24.316
2017	24.674	25.387	25.679	24.840	24.410	24.096	23.594	23.412	23.626	23.507	23.359	24.291	24.240
2018	23.920	25.196	24.882	24.599	24.175	24.062	23.510	23.692	23.770	23.403	24.132	23.725	24.089
**X**	23.735	24.761	24.751	24.361	23.865	23.394	22.950	23.022	22.999	22.827	23.162	23.337	23.597
