# Supplementary material for: Reproductive seasonality in the Baka Pygmies, environmental factors and climatic changes
Source: PLoS One. 2022 Mar 8;17(3):e0264761. doi: 10.1371/journal.pone.0264761 (PMC8903253; doi:10.1371/journal.pone.0264761)
Supplement: S3 Table — (PDF) [file pone.0264761.s004.pdf]

S3 Table. Fertility rates per month and year, using monthly numbers of births and yearly numbers of women

	2007	2008	2009	2010	2011	2012	2013	2014	2015	2016	2017	2018
**January**	0.01136	0.02752	0.02581	0.03205	0	0.01176	0.01351	0	0.01818	0.01802	0.02899	0
**February**	0.02273	0.02752	0.01290	0.02564	0.02703	0.00588	0	0.00559	0	0.00901	0.00725	0.01143
**March**	0.02273	0.01835	0.01935	0.03205	0.00676	0.01765	0.01351	0.02235	0.00606	0.05405	0.01449	0
**April**	0.01136	0.05505	0.03226	0.00641	0.02027	0.02353	0	0.02235	0.02424	0.03604	0.01449	0.01714
**May**	0.03409	0.03670	0.01935	0.00641	0	0.03529	0.01351	0.02235	0.02424	0.05405	0.00725	0.02286
**June**	0	0.06422	0.01935	0.01282	0.01351	0.01176	0.01351	0.03352	0.00606	0.00901	0.02899	0.02286
**July**	0.01136	0.00917	0.05161	0.01923	0.00676	0.01176	0.02703	0.03352	0.01818	0.02703	0.01449	0.00571
**August**	0.02273	0.01835	0.00645	0.00641	0.02027	0.00588	0.01351	0	0.00606	0.01802	0.02899	0
**September**	0.01136	0.03670	0.01935	0.02564	0.02027	0.01176	0.00676	0.01676	0.03636	0.05405	0.01449	0
**October**	0.03409	0	0.01935	0.01282	0.02027	0.02941	0.00676	0.00559	0.00606	0.02703	0.02174	0.01143
**November**	0.01136	0.03670	0.00645	0.03205	0.01351	0.01176	0.02703	0.00559	0.03030	0.05405	0.00725	0
**December**	0.01136	0.01835	0.01290	0.02564	0.02027	0.01176	0.02027	0.02793	0.00606	0.00901	0.00725	0
