# Supplementary material for: Reproductive seasonality in the Baka Pygmies, environmental factors and climatic changes
Source: PLoS One. 2022 Mar 8;17(3):e0264761. doi: 10.1371/journal.pone.0264761 (PMC8903253; doi:10.1371/journal.pone.0264761)
Supplement: S1 Fig — For each set of values (A and B), two types of graph were produced, boxplots (left) to show the raw data distribution and locally weighted polynomial plots (right) generated with the ‘locally.weighted.polynomial’ of the SiZer library in ‘R’, to show smoothed data. (PDF) [file pone.0264761.s005.pdf]

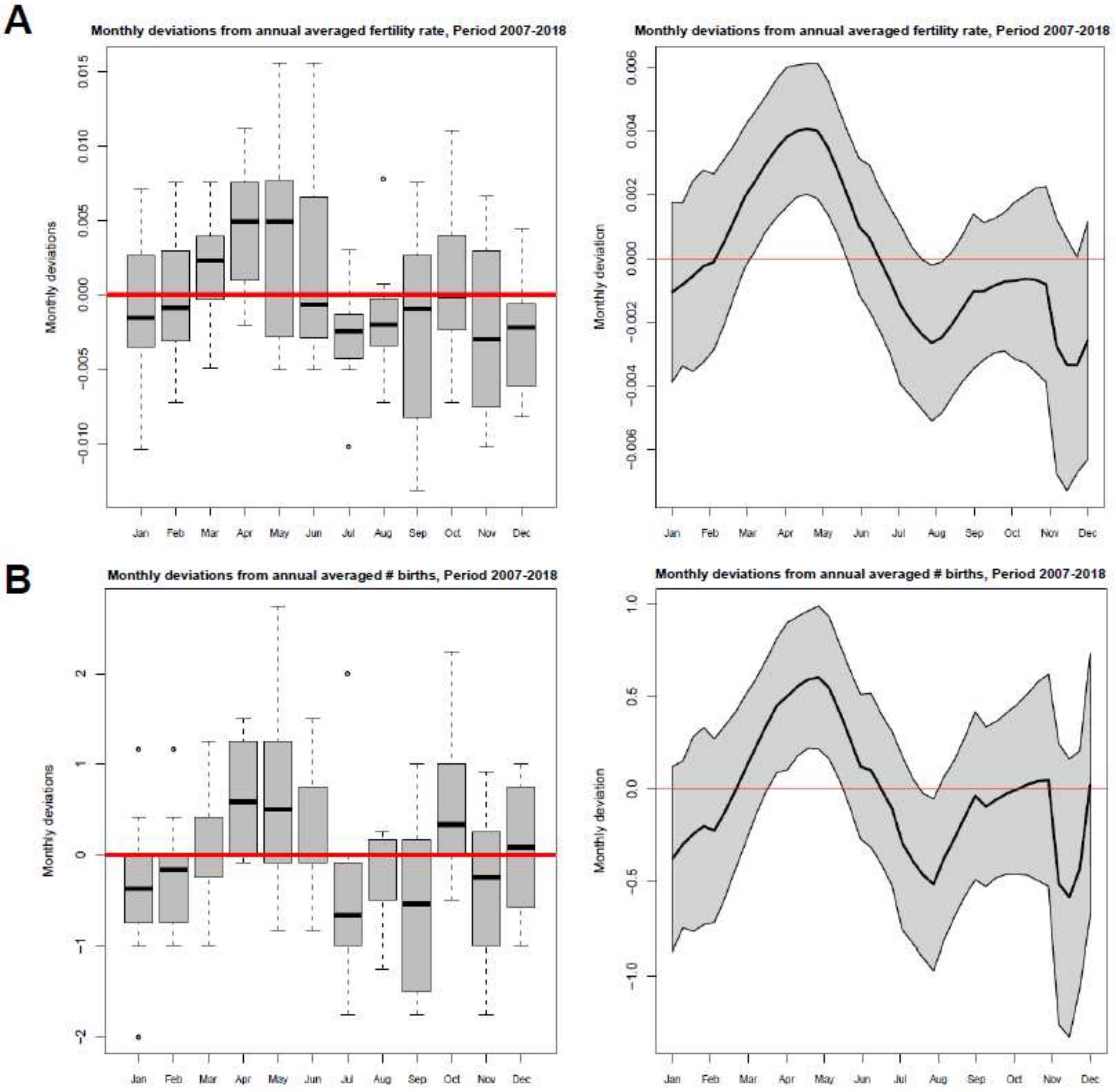


**S1 Fig**. **Monthly deviations from annual averaged fertility rate (A) and from the annual averaged number of births (B) for the 2007-2018 period**. For each set of values (A and B), two types of graph were produced, boxplots (left) to show the raw data distribution and locally weighted polynomial plots (right) generated with the 'locally.weighted.polynomial' of the SiZer library in 'R', to show smoothed data.
