# Supplementary material for: Reproductive seasonality in the Baka Pygmies, environmental factors and climatic changes
Source: PLoS One. 2022 Mar 8;17(3):e0264761. doi: 10.1371/journal.pone.0264761 (PMC8903253; doi:10.1371/journal.pone.0264761)
Supplement: S2 Fig — The red line stands for the sinusoidal regression of wavelength of 2π/6 with p = 0.02272 (a = -0.04412 and b = -0.33962). The number of births per year (showing that it varies from 16 to 55 recorded births) and the raw data variations in the number of births from month to month analyzed by harmonic regression confirm that birth seasonality is significantly bimodal. (PDF) [file pone.0264761.s006.pdf]

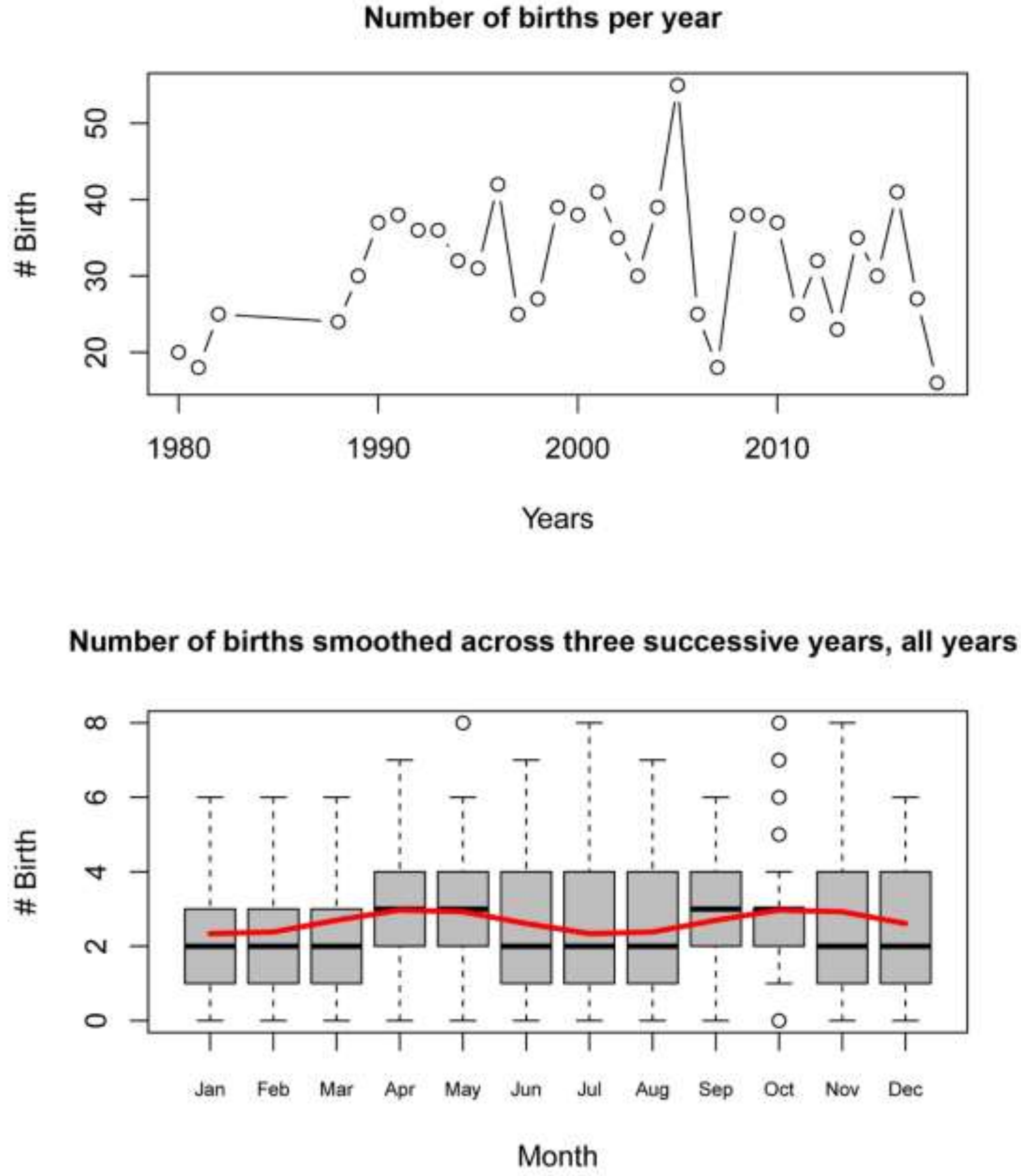

S2 Fig. Number of births per year (upper panel) and boxplot of the number of births across months (lower panel). The red line stands for the sinusoidal regression of wavelength of 2π/6 with p=0.02272 (a=-0.04412 and b=-0.33962). The number of births per year (showing that it varies from 16 to 55 recorded births) and the raw data variations in the number of births from month to month analyzed by harmonic regression confirm that birth seasonality is significantly bimodal.
