# Supplementary material for: Reproductive seasonality in the Baka Pygmies, environmental factors and climatic changes
Source: PLoS One. 2022 Mar 8;17(3):e0264761. doi: 10.1371/journal.pone.0264761 (PMC8903253; doi:10.1371/journal.pone.0264761)
Supplement: S3 Fig — Months when Dev.Birth is at a minimum between November of the previous year and February (i.e., ‘minimum 1’, down-pointing triangle) and at a maximum between the two minimums (i.e., ‘maximum 1’, up-pointing triangle). The linear regression lines have non-significant coefficients (p-value>0.1). (PDF) [file pone.0264761.s007.pdf]

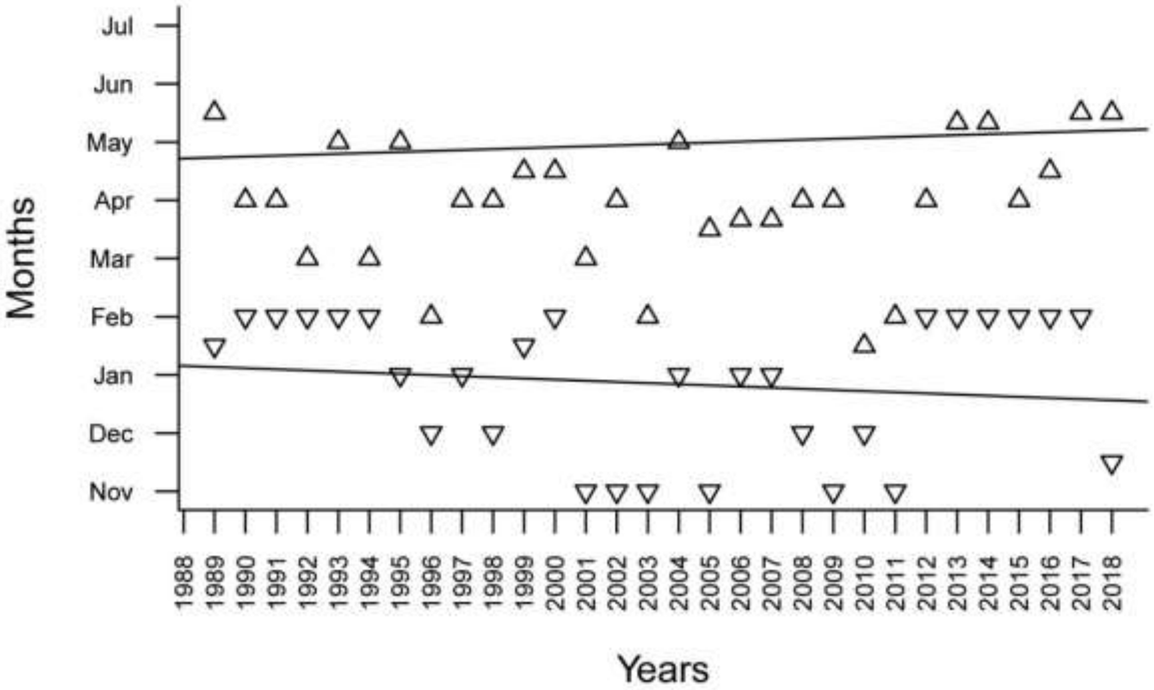

S3 Fig. Extremes of birth month deviations for the first half of the year. Months when *Dev.Birth* is at a minimum between November of the previous year and February (i.e., ‘minimum 1’, down-pointing triangle) and at a maximum between the two minimums (i.e., ‘maximum 1’, up-pointing triangle). The linear regression lines have non-significant coefficients (p-value>0.1).
