# Supplementary material for: Reproductive seasonality in the Baka Pygmies, environmental factors and climatic changes
Source: PLoS One. 2022 Mar 8;17(3):e0264761. doi: 10.1371/journal.pone.0264761 (PMC8903253; doi:10.1371/journal.pone.0264761)
Supplement: Attachment — Submitted filename: Review.pdf [file pone.0264761.s009.pdf]

Seasonality of birth in the Baka Pygmies, environmental factors and climatic changes

Signed review by Ed Hagen

8/20/2021

Summary

Rozzi et al. analyze a remarkable data set of 34 years of births among a small Baka population. They find a bimodal seasonal pattern, with births peaking every 6 months. This pattern plausibly relates to seasonal climate variation – precipitation and temperature – as well as subsistence practices, and has been shifting over the years as climate has shifted.

I am not a demographer, nor do I have much experience analyzing time series data, so I can't evaluate the appropriateness of the statistical analyses. I therefore review this manuscript as a behavioral ecologist interested in hunter-gatherer demography, and one who has worked with a similar group of Congo Basin foragers, the Aka, who live in the neighboring Central African Republic (CAR).

This is an interesting, well-written paper. The introduction provides a nice overview of seasonality in births among humans and non-human animals for those who are new to this topic, as well as a nice overview of the Baka. The authors' findings of a bimodal seasonal pattern of births and its connection to precipitation and temperature is worth publishing. The Discussion section hits all the main points that I would want to see, e.g., connections of seasonal births to seasonal subsistence practices. One question here: the Aka have a caterpillar season, which brings in substantial food and cash. Do the Baka have the same?

The only concern I had was that the data are pretty noisy, as the authors admit. They therefore undertook several smoothing steps to reveal the bimodal pattern (e.g., median smoothing). I was therefore curious to look at the raw data myself. I imported their Table 1 into R. My very simple analyses confirm a strong bimodal pattern when aggregating data by month across all years. But my analyses also raised a few questions that the authors might want to address. (I emphasize again that I have no expertise in demography or time series analysis.)

Import data

I was able to convert Table 1 from PDF to Excel using an online tool from Adobe. It would be nice if the authors provided an Excel spreadsheet in their SI.

```
library(readxl)
library(tidyverse)

# I used Adobe's online pdf-2-excel converter to extract this table from the ms.
# https://www.adobe.com/acrobat/online/pdf-to-excel.html
# I did not import the marginal row or column totals.
# I spot-checked several values against the ms for accuracy, but did not check every value.
d <-
```

```
  read_excel('Baka_births.xlsx', skip = 2, n_max = 34) %>%
  dplyr::select(Year, Jan:Dec)

knitr::kable(head(d))
```

Year	Jan	Feb	Mar	Apr	May	Jun	Jul	Aug	Sep	Oct	Nov	Dec
1980	3	3	1	2	2	1	0	1	3	1	0	3
1981	1	1	1	0	0	1	0	2	3	2	2	5
1982	0	3	1	4	1	2	5	1	2	3	1	2
1988	0	2	0	2	4	1	0	3	2	3	3	4
1989	2	3	3	3	4	1	2	2	0	7	2	1
1990	2	1	4	7	4	2	3	4	1	2	1	6

Converting the table to a time series

```
d2 <-
  d %>%
  set_names(c('Year', 0:11)) %>% # rename months to their numeric value
  pivot_longer("0":"11", names_to = 'Month', values_to = 'Births') %>%
  mutate(
    Year = as.numeric(Year),
    Year = ifelse(Year <= 1982, Year + 5, Year), # Remove gap between 1982 and 1988
    Month = as.numeric(Month),
    Time = Year + Month/12 # Convert month/year to decimal Time
  )

knitr::kable(head(d2))
```

Year	Month	Births	Time
1985	0	3	1985.000
1985	1	3	1985.083
1985	2	1	1985.167
1985	3	2	1985.250
1985	4	2	1985.333
1985	5	1	1985.417

Plot raw timeseries

The raw data are quite noisy, and a bimodal pattern is not readily apparent.

```
ggplot(d2, aes(Time, Births)) + geom_line() + scale_x_continuous(breaks = seq(1985, 2018, 5))
```

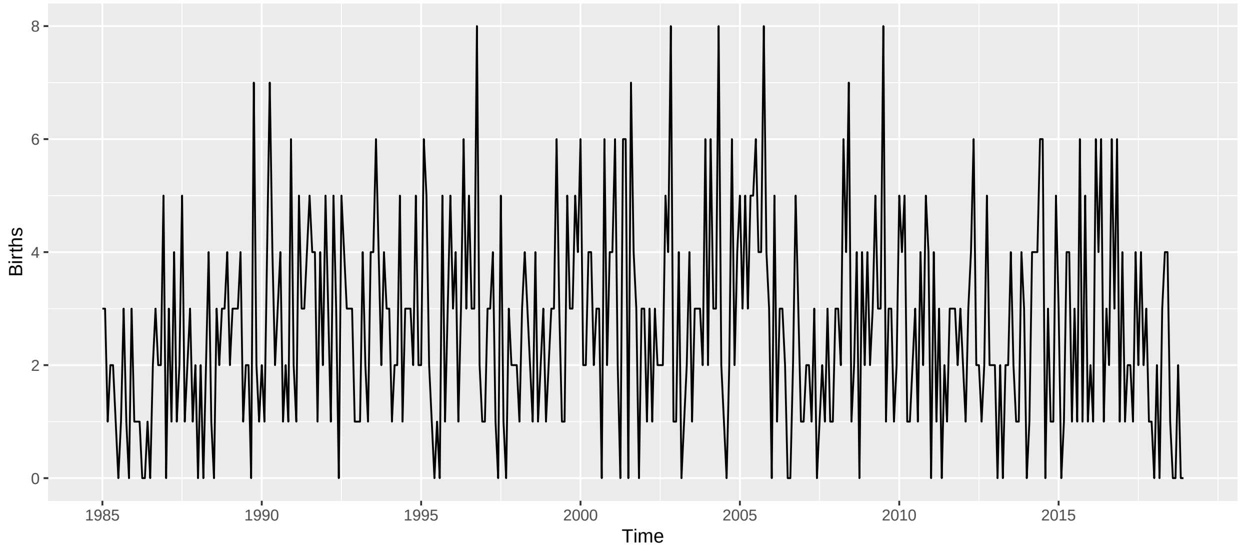

Total births by month across all years

Totalling births by month across all years, however (the bottom row of the authors' Table 1), reveals a strong bimodal pattern.

```
d2 %>%
  group_by(Month) %>%
  summarise(
    TotalBirths = sum(Births)
  ) %>%
  ggplot(aes(Month, TotalBirths)) + geom_line() + scale_x_continuous(breaks = 0:11) # Jan: 0, Dec: 11
```

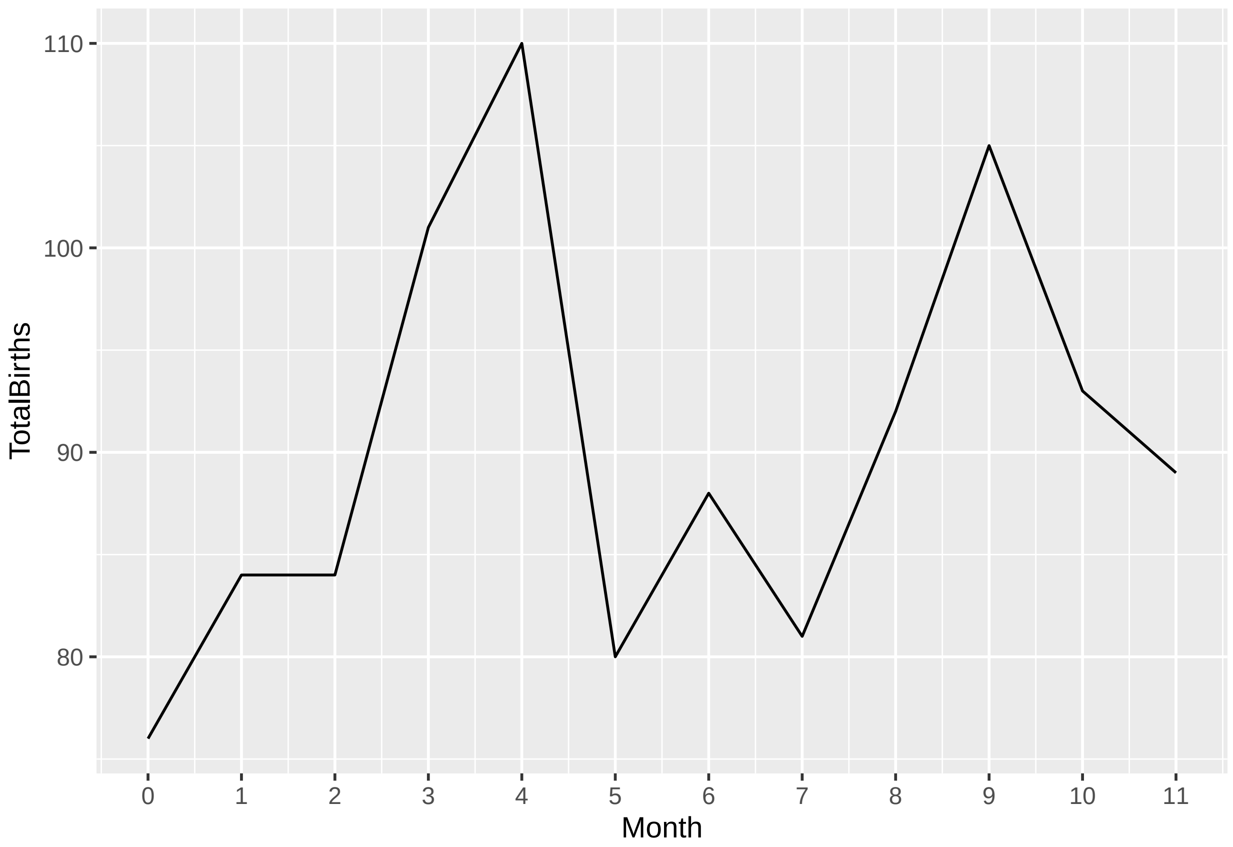

Total births by month for 2001 and earlier

Restricting analysis to the first half of the data, however, does not reveal a clear bimodal pattern, although there are peaks that correspond to the bimodal peaks seen in the complete data.

```
d2 %>%
  dplyr::filter(Year <= 2001) %>%
  group_by(Month) %>%
  summarise(
    TotalBirths = sum(Births)
  ) %>%
  ggplot(aes(Month, TotalBirths)) + geom_line() + scale_x_continuous(breaks = 0:11) # Jan: 0, Dec: 11
```

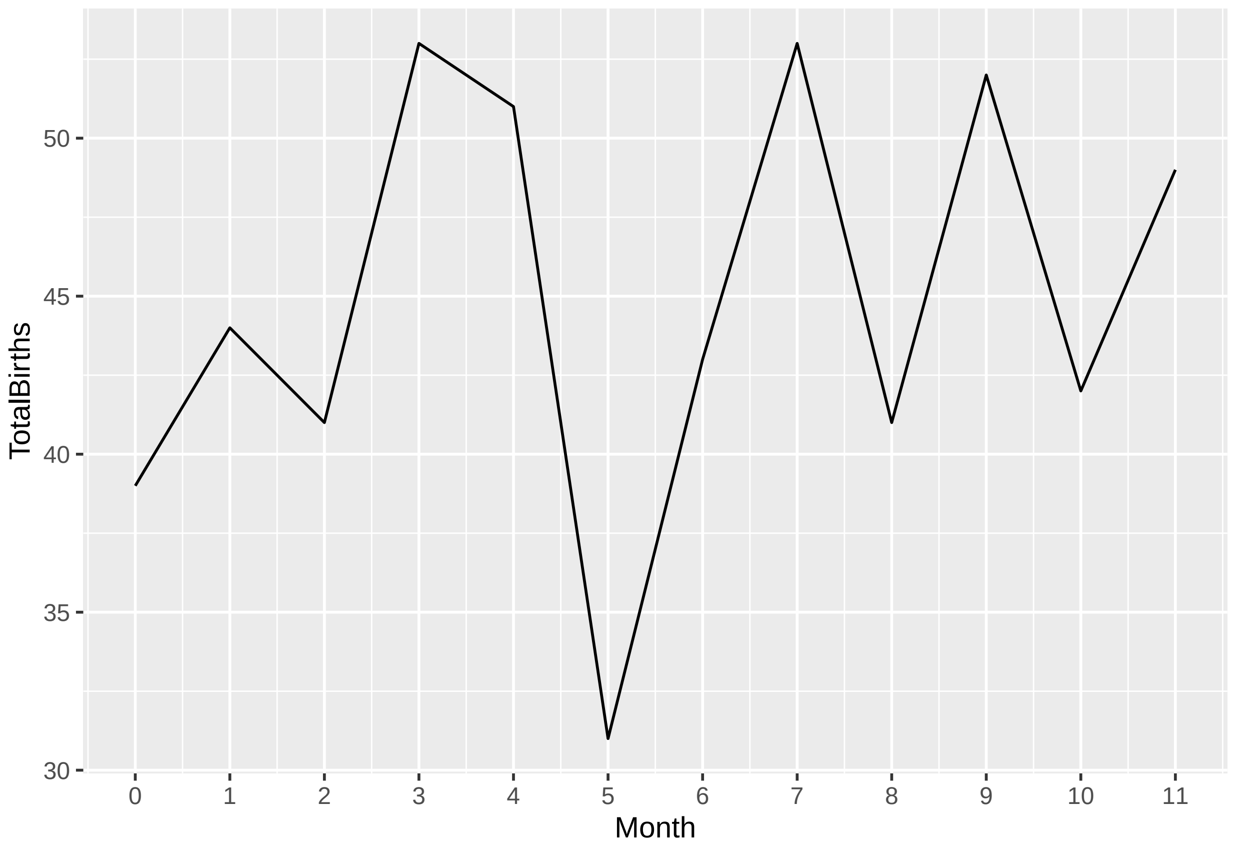

Total births by month for 2002 and later

The bimodal pattern is clear in the second half of the data, though.

```
d2 %>%
  dplyr::filter(Year > 2001) %>%
  group_by(Month) %>%
  summarise(
    TotalBirths = sum(Births)
  ) %>%
  ggplot(aes(Month, TotalBirths)) + geom_line() + scale_x_continuous(breaks = 0:11) # Jan: 0, Dec: 11
```

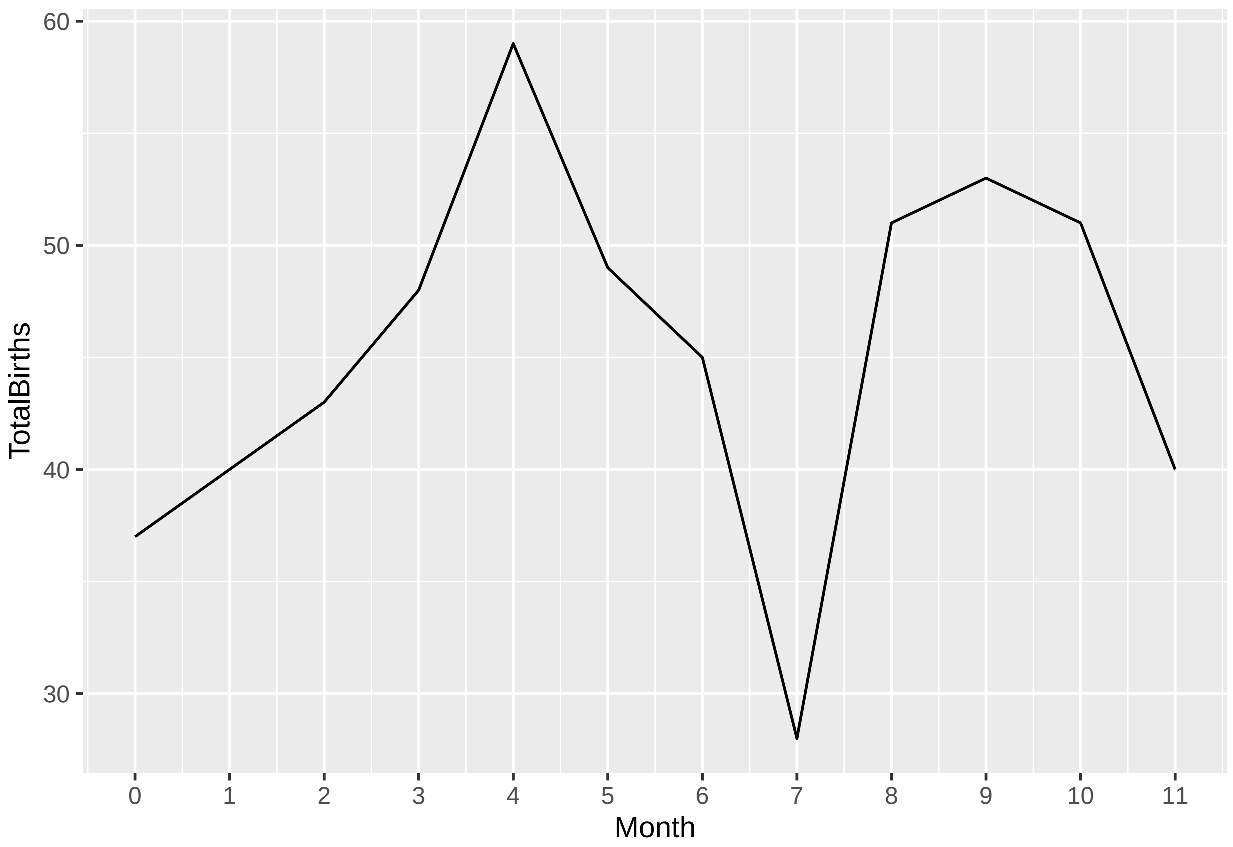

Power spectrum (naive)

Interestingly, a naive power spectrum analysis reveals a strong peak at 4 cycles per year. The authors used harmonic regression to test for either 1 peak or 2 peaks a year. This result suggests that there might instead be 4 peaks. But I'm not an expert in time series analysis, so perhaps this result is an artifact of some sort (or maybe I'm doing something wrong). I leave it to the authors to consider if they should investigate.

```
m <- spectrum(d2$Time, log="no", plot=F)
plot(m$freq*12, 2*m$spec, xlab="Frequency", ylab = 'Spectral density', type="l")
```

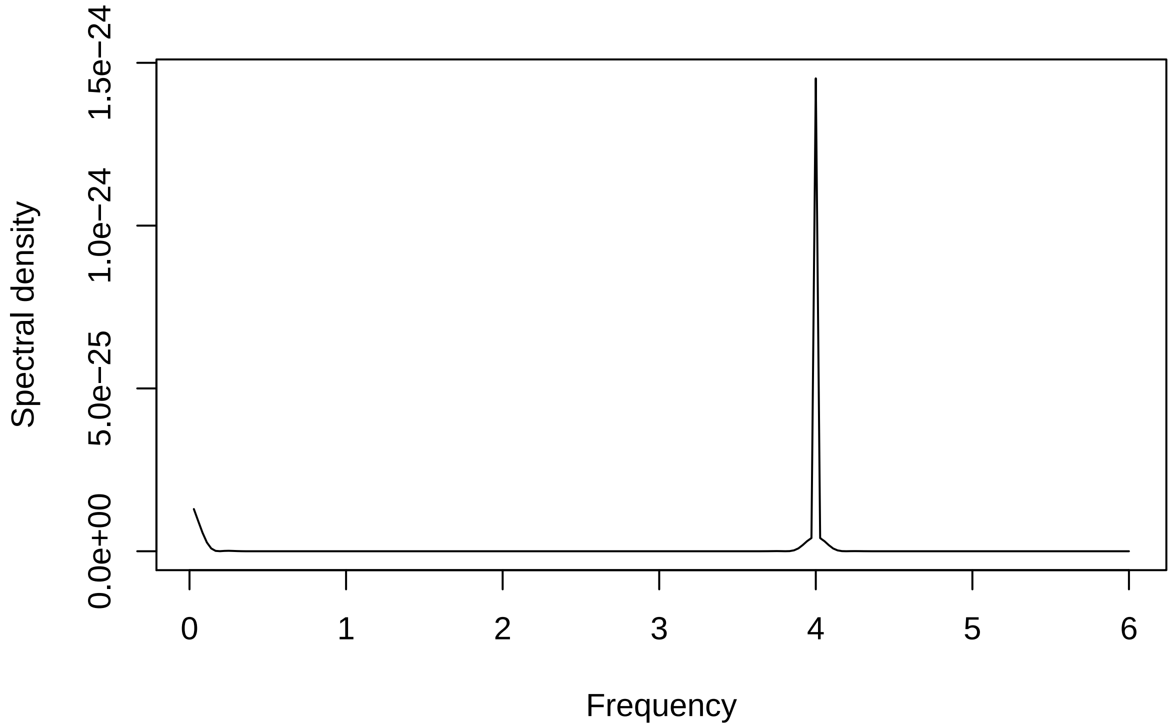

Power spectrum for 1988-2018 only

There is a 5 year gap in the original data, so here I just used the data after the gap. The pattern is the same.

```
m <- spectrum(d2$Time[d2$Year>=1988], log="no", plot=F)
plot(m$freq*12, 2*m$spec, xlab="Frequency", ylab = 'Spectral density', type="l")
```

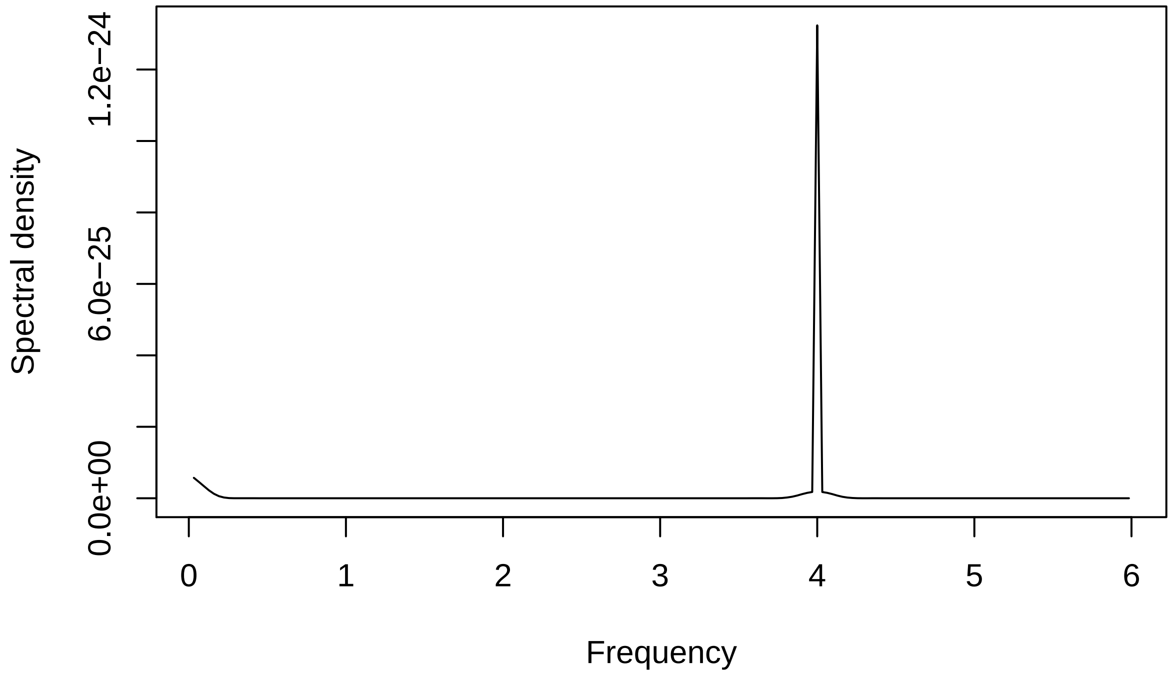

Minor

Order of authors on SI differs from order on ms.
