# Supplementary material for: Reproductive seasonality in the Baka Pygmies, environmental factors and climatic changes
Source: PLoS One. 2022 Mar 8;17(3):e0264761. doi: 10.1371/journal.pone.0264761 (PMC8903253; doi:10.1371/journal.pone.0264761)
Supplement: Attachment — Submitted filename: BirthSeasonalityBakaPiqueFandinoetal_mc.docx [file pone.0264761.s008.docx]

**Seasonality of birth in the Baka Pygmies, environmental factors and climatic changes**

Laura Piqué-Fandiño^1^, Sandrine Gallois^2^, Samuel Pavard^1¶^ and Fernando V. Ramirez Rozzi^1,3^ ^¶*^

^1^UMR 7206 Ecoanthropology, MNHN, CNRS, UP, Paris, France.

^2^ Universitat oberta de Catalunya, Barcelona, Spain.

^3^EA 2496, UP, Faculté de Chirurgie Dentaire, Montrouge, France

¶ These authors supervised this study equally

**Corresponding author:** Fernando V. Ramirez Rozzi, [fernando.ramirez-rozzi@mnhn.fr](mailto:fernando.ramirez-rozzi@mnhn.fr)

**Keywords:** Hunter-gatherer, human fertility, mobility, socio-cultural practices, birth pattern

**Abstract**

Birth seasonality is a phenomenon common to human and animal populations and driven by, among others, climatic variables. Given the currently changing climate and its impacts on both the environment and human lives, the question arises of its potential effects on birth seasonality. Because indigenous people, like hunter-gatherers, are among the most vulnerable human societies, we need to further explore the impacts of these changes on their lives and health. No study has specifically explored the seasonality of births among hunter-gatherers and how current climate change might affect this phenomenon. In this study we addressed birth seasonality in the Baka Pygmy hunter-gatherer society living in African rain forests. Since birth seasonality can be linked to weather patterns, we explore this possibility. However, climatic variables driving weather patterns have changed over the years, so we assessed whether this has influenced the Baka birth pattern. Based on 34 years of written birth records and oral questionnaires from 13 years of systematic fieldwork, we observed a bimodal birth pattern with two birth peaks at 6-month intervals, which relates to a short interbirth interval. Our results demonstrate that birth seasonality is significantly linked to precipitation and temperature. Changing weather patterns appear to be affecting the birth seasonality in the Baka, suggesting that attention needs to be given to the influence of global climate change on forager societies.

**Introduction**

Studying human birth seasonality is of crucial importance because birth months have significant consequences for many demographic aspects such as infant mortality [e.g. 1, 2], maternal performance [3], adult height [4], lifespan [5, 6] and susceptibility to late-onset diseases [e.g. 7-9]. This is a classic issue in demography [e.g. 10, 11], whose significance has been demonstrated in the vast majority of populations from the broad national scale [e.g. 12] to the local scale [13], but with considerable differences in pattern, magnitude and timing [e.g. 12].

Seasonal birth patterns are observed not only in human populations, but also in other mammal species such as rodents and artiodactyls [14], wild dogs [15] and primates [16-18]. Heldstab et al. [18] and Kiltie [19] suggest that it would not be adaptive for mammals with a slow life history to have restricted birth seasons, since their long reproductive cycles would not fit into the short time window for optimal environmental conditions. However, although great apes do not have restricted birth seasons, they do show seasonal distributions in the frequency of births [20]. For instance, in chimpanzees conceptions tend to occur during periods of increased food supply [16] while in free-ranging orangutan populations, it is related to the mast fruiting season and to body condition [21].

While food supply and body condition seem to be the cause of seasonality in other primate species, in humans, the biological environmental and socio-cultural determinants of birth seasonality are still unclear and are highly variable among populations. The different determinants of seasonality in humans fall into three main categories related to social, climatic and energetic factors [20]. Firstly, seasonal socio-cultural aspects such as marriages, holidays, periods of work and religious celebrations [e.g. 22-24] have been shown to shape the distribution of births over a year in several populations [25]. Secondly, environmental or climatic determinants such as temperature [e.g. 26, 27] and rainfall [e.g. 13, 28-30] have also been shown to shape birth seasonality in several societies. For instance, rates of conception are lower in extreme temperatures [e.g. 26, 27], which may be due to a lower frequency of intercourse [e.g. 29, 31] or to biological factors affecting conception [e.g. deterioration of semen quality during warm months, 32]. Rainfall prior to conception is associated with births both directly and indirectly. Rainfall has also proved to shape the birth seasonality through differential food availability [13, 29, 33] or increased incidence of infectious diseases, such as malaria, which may in turn depress fecundability [28, 30]. Additionally, rainfall and temperature are associated with many human subsistence activities that can modulate the frequency of intercourse as well as the population's perception of the auspiciousness of having a child. As regards the latter factor, weather conditions at the time of birth, not conception, may therefore also influence birth seasonality [34], as parents might choose to give birth in periods with better climatic conditions that improve food availability and reduce the likelihood of diseases. Other environmental characteristics such as latitude have been found as determinants of the timing of the human birth peak, with peaks coming earlier in the year at locations further from the equator [35]. Finally, there are the energetic determinants. Because pregnancy is a process that demands high energy expenditure, conceptions must be concentrated in periods when the energy balance is positive [33]. Thus, a good nutritional status has been indicated as a key factor in higher conception rates [12, 33, 36].

Even though birth seasonality is a classic topic of investigation in anthropological and historical demography [e.g. 6, 11, 25, 29], only few studies have investigated the presence and causes of birth seasonality in hunter-gatherer societies [13, 37, 38]. This is quite surprising considering that these societies depend on a very close relationship with the environment for their culture and livelihood (including food resources and medicine), and can thus be expected to be more directly influenced by climatic variables (i.e. rainfall and temperature). The small amount of literature on hunter-gatherers might be explained by a lack of access to accurate data. Unlike studies in agricultural and industrialized societies, studies of demographic aspects such as birth seasonality in hunter-gatherers raise particular challenges in terms of data quality and quantity. The lack of chronology and the absence of written records introduce a bias whose magnitude is unknown in most cases. Bailey et al. [13] did not observe any birth pattern in the Efe Pygmies from the Ituri forest. Among the San from the Kalahari, Wilmsen [37, 39] found that precipitation is the main factor influencing the birth seasonality. On the other hand, Hill and Hurtado [38], in their study on the Ache from Paraguay, did not relate birth seasonality directly to environmental conditions but rather to the energy balance, since they found that fewer conceptions correlated with lower body weights. Therefore, it seems that environmental variables, and more specifically climatic variables, might be related to birth seasonality among hunter-gatherers. However, we still need further study to better understand birth patterns in these societies.

The fact that climatic variables might relate to birth patterns and birth seasonality in human populations raises another topical issue. The cascading effects of climate change in these last decades are not only affecting the physical and biological components of the planet (such as sea level, ice retreat, freshwater availability and biodiversity loss), but also the means of subsistence, health and well-being of human populations [40, 41]. For instance, Philibert et al. [30] have observed that recent climatic changes have influenced birth seasonality in industrialized societies. Indigenous peoples and local communities, such as hunter-gatherers, are indeed considered to be the populations most vulnerable to climate change because of their overall marginalization, lack of land tenure and poor access to health services and justice systems [42]. Therefore, considering the importance of birth seasonality for demographic and health issues, it is worth investigating whether climate change might be impacting the birth pattern among these populations.

This study therefore has three main aims. The first is to assess the presence and pattern of birth seasonality in the Baka Pygmies who live in the African rainforest. Since previous studies of the Ache in the rainforests of eastern Paraguay have demonstrated birth seasonality [28], we expect the Baka to show birth seasonality as well. Recent studies on growth and life history variables in the Baka Pygmies in Cameroon [43, 44] have shown that the median and average interbirth interval (IBI) is 2.5 years. Since the IBI is 2.5 years, successive siblings are born at two different times in the year. If a peak marks a seasonal birth pattern in the Baka, we would expect a second peak six months later and thus a two-peak pattern of birth seasonality. Secondly, we aim to explore whether climatic variables, such as temperature and rainfall, might affect the birth pattern in the Baka. Several previous studies have found a link between weather variables and birth seasonality, in particular conception in periods of positive energy balance due to better ovarian function [e.g. 16, 21, 26, 28, 29, 38]. If this is true, we would expect a conception peak in the Baka Pygmies in the dry season when the overall availability of wild food is greater [i.e. 45, 46]. Finally, if climatic and birth patterns are related, our third aim is to investigate the relationship between global climate change and local birth patterns. Because temperatures are increasing worldwide and rainfall patterns are changing in different parts of the planet due to climate change, we expect to see variations in both variables over the last 30 years, with an effect on reproductive seasonality [e.g. 11, 26, 47]. This study is based on 13 years of systematic and regular fieldwork and on 34 years of birth records, the largest number of such records ever obtained (n=1803) for a hunter-gatherer population ([13] n=147, [37] n=221).

**Materials and Methods**

**Materials**

The Baka Pygmies

The so-called Pygmy populations are semi-nomadic hunter-gatherers living in the equatorial rain forests of Africa. They have an independent evolutionary history which split from non-Pygmy populations around 60000BP [48]. Two clusters of Pygmy groups then split out around 2800 BP [49]: one in West Africa (the Twa, Kola, Bongo, Koya, Aka and Baka groups) and the other in East Africa (the Aka, Sua, Mbuti/Efe groups and the BaTembo). African Pygmies grow to an average adult stature of <155cm [50]. The genetic bases of this very specific phenotype are polygenic. They include genes from the growth hormone axis as well as genomic and gene regions that relate to the traits themselves [51-53]. Pygmy morphology has long been interpreted as an adaptation to forest life [50, 51] in an extremely humid and dense environment [54].

Among these different Pygmy groups, the Baka have a geographical distribution comprising south-eastern Cameroun, north-eastern Gabon and north-western Congo. Based on genetic evidence and despite their semi-nomadic lifestyle, the pattern of Baka dispersal is highly localized, with a maximum parent-offspring dispersal of approximately 60km [55]. The climate in the Guineo-Congolese evergreen forest [56-58] where they became established is typical of inland areas near the equatorial zone, with year-round moderate to high temperatures (monthly mean ~23ºC) and seasonal rainfall with two wet and two dry seasons annually and average monthly precipitation of <1540mm [59-60]. The rainy seasons are characterized by short periods of torrential rainfall [61]. The months and duration of the seasons may vary slightly, by more or less one month, depending on the specific geographical area.

The habitat where the Baka live provides them with food all year around, from both foraging activities (hunting, gathering and fishing) and agriculture. The Baka do not present nutritional deficiencies [62] and no episode of starvation or famine has been recorded [44]. However, there are seasons when specific foods are scarcer or more abundant [45, 63]. The fluctuations in the availability of wild food resources and in agricultural work imply that Baka subsistence activities vary according to season [64]. Overall, agricultural tasks are conducted all year long, but more intense work is needed when digging new plots, in both dry seasons, and when planting groundnuts (around April). Foraging patterns also vary, with some important periods of more intense activity. For instance, gathering wild or “bush” mangoes (Irvingiaceae family), mostly from June to August [65], is a fundamental activity and a key period in the Baka calendar. During this time, all adult men and women, as well as children, spend most of their time gathering the fruit [45, 65]. The intensity of gathering is such that bush mango reserves are generally depleted by the end of July or the beginning of August (FRR, personal observation). Hunting activities, mainly by the men, are more intense during both rainy seasons (September to December and March to June). Fishing, mainly done by women, is more frequent during the dry seasons, from December to March and from July to August. Moreover, even if women and men invest more time in fishing and hunting respectively, the evidence shows that women also spend time on hunting and men on fishing [66, 67].

The study took place in Moange Le Bosquet (3º05’07’’N 14º03’45’’E), a Baka Pygmy village in south-eastern Cameroon. Founded in 1973 around a Catholic mission, Le Bosquet has a medical centre run by nuns with medical training whose community was established when the village was founded, to provide health care and keep records of births in the Baka community. The Baka from Le Bosquet share their life between the village itself and camps in the middle of the forest, about 15 km away. The births of Baka children are registered in the centre's records even when the families are spending time in their forest camps, since travel between the camps and the village is frequent. Birth records with both parents identified were available to us from 1980 to 1982 and from 1988 until now. According to several censuses carried out by the nuns and FRR over the last 13 years, the number of Baka individuals in Le Bosquet has remained more or less constant since 1973 at around 800 individuals, divided into approximately 230 households. Thanks to the nuns’ and our censuses, we know that approximately half of the households register births in the medical centre. This subpopulation in Moange Le Bosquet makes up the subject of our study. It comprises 406 households, which is higher than the total number of households in Le Bosquet in any given year because the birth records over 13 years include many households who were living in Le Bosquet but moved out after some years. The constant number of inhabitants in Le Bosquet since its foundation implies that the number of families moving in and out is more or less the same.

Prior informed consent was obtained from all participants and the study was carried out with the approval of the chief of the Baka community. All methods are non-invasive and were carried out in accordance with relevant guidelines and regulations. This study obtained approval from the Centre National de la Recherche Scientifique and Agence National de la Recherche (France) and from the Institut de Recherche et Développement, and was carried out under an international agreement between the Institut de Recherche pour le Développement (IRD) and the Ministry of Scientific Research and Technology of Cameroon and the National Committee of Ethics for Research for Human Health of Cameroon (2018/06/1049/CE/CNERSH/SP).

Data

The data comprise a total of 1083 records of births over 34 years (from 1980 to 1982 and from 1988 to 2018) (Table 1). These records include the names of both parents and the exact date of birth (day, month and year; only 14 records lack the exact day). The number of births per year ranges from 16 to 55 with an average of 31.85 (SD=8.4).  The number of women of child-bearing age (15 to 55 [44]) present in the village every year is known from 2007 to 2018 (44) (Table 1). The field studies conducted once or twice every year in Moange le Bosquet from 2007 until 2019 included questionnaires about family composition.

In order to assess the potential link between environmental conditions and the birth pattern, we used data from the ERA5 report provided by the European Centre for Medium-Range Weather Forecasts (ECMWF, https://www.ecmwf.int). ERA5 provides hourly estimates of a large number of climate variables, covering the Earth on a 30km grid. ERA5 compiles vast amounts of historical observations into global estimates using advanced modelling and data assimilation systems [68]. Data available from the closest weather station to Moange le Bosquet are from the village of Messok, 35 km to the east. The wet seasons occur in March-May and September-November and the dry seasons in December-February and June-August. The meteorological data include precipitation and temperature from 1978 to 2018.

**Table 1. Number of births by month and by number of women present in Le Bosquet**

|  | **Number of birth** | | | | | | | | | | | |  | **Women** |
| --- | --- | --- | --- | --- | --- | --- | --- | --- | --- | --- | --- | --- | --- | --- |
| Year | Jan | Feb | Mar | Apr | May | Jun | Jul | Aug | Sep | Oct | Nov | Dec | Total |  |
| 1980 | 3 | 3 | 1 | 2 | 2 | 1 | 0 | 1 | 3 | 1 | 0 | 3 | 20 |  |
| 1981 | 1 | 1 | 1 | 0 | 0 | 1 | 0 | 2 | 3 | 2 | 2 | 5 | 18 |  |
| 1982 | 0 | 3 | 1 | 4 | 1 | 2 | 5 | 1 | 2 | 3 | 1 | 2 | 25 |  |
| 1988 | 0 | 2 | 0 | 2 | 4 | 1 | 0 | 3 | 2 | 3 | 3 | 4 | 24 |  |
| 1989 | 2 | 3 | 3 | 3 | 4 | 1 | 2 | 2 | 0 | 7 | 2 | 1 | 30 |  |
| 1990 | 2 | 1 | 4 | 7 | 4 | 2 | 3 | 4 | 1 | 2 | 1 | 6 | 37 |  |
| 1991 | 2 | 1 | 5 | 3 | 3 | 4 | 5 | 4 | 4 | 1 | 4 | 2 | 38 |  |
| 1992 | 5 | 3 | 1 | 5 | 3 | 0 | 5 | 4 | 3 | 3 | 3 | 1 | 36 |  |
| 1993 | 1 | 1 | 4 | 2 | 1 | 4 | 4 | 6 | 4 | 2 | 4 | 3 | 36 |  |
| 1994 | 3 | 1 | 2 | 2 | 5 | 1 | 3 | 3 | 3 | 2 | 5 | 2 | 32 |  |
| 1995 | 2 | 6 | 5 | 2 | 1 | 0 | 1 | 0 | 5 | 1 | 3 | 5 | 31 |  |
| 1996 | 3 | 4 | 1 | 3 | 6 | 3 | 5 | 3 | 3 | 8 | 2 | 1 | 42 |  |
| 1997 | 1 | 3 | 3 | 4 | 1 | 0 | 5 | 1 | 0 | 3 | 2 | 2 | 25 |  |
| 1998 | 2 | 1 | 3 | 4 | 3 | 2 | 1 | 4 | 1 | 2 | 3 | 1 | 27 |  |
| 1999 | 2 | 3 | 3 | 6 | 3 | 1 | 1 | 5 | 3 | 3 | 5 | 4 | 39 |  |
| 2000 | 6 | 2 | 2 | 4 | 4 | 2 | 3 | 3 | 0 | 6 | 2 | 4 | 38 |  |
| 2001 | 4 | 6 | 2 | 0 | 6 | 6 | 0 | 7 | 4 | 3 | 0 | 3 | 41 |  |
| 2002 | 3 | 1 | 3 | 1 | 3 | 2 | 2 | 2 | 5 | 4 | 8 | 1 | 35 |  |
| 2003 | 1 | 4 | 0 | 1 | 2 | 4 | 1 | 3 | 3 | 3 | 2 | 6 | 30 |  |
| 2004 | 2 | 6 | 3 | 3 | 8 | 2 | 1 | 0 | 2 | 6 | 2 | 4 | 39 |  |
| 2005 | 5 | 3 | 5 | 3 | 5 | 5 | 6 | 4 | 4 | 8 | 4 | 3 | 55 |  |
| 2006 | 0 | 5 | 1 | 3 | 3 | 2 | 0 | 0 | 2 | 5 | 3 | 1 | 25 |  |
| 2007 | 1 | 2 | 2 | 1 | 3 | 0 | 1 | 2 | 1 | 3 | 1 | 1 | 18 | 88 |
| 2008 | 3 | 3 | 2 | 6 | 4 | 7 | 1 | 2 | 4 | 0 | 4 | 2 | 38 | 109 |
| 2009 | 4 | 2 | 3 | 5 | 3 | 3 | 8 | 1 | 3 | 3 | 1 | 2 | 38 | 155 |
| 2010 | 5 | 4 | 5 | 1 | 1 | 2 | 3 | 1 | 4 | 2 | 5 | 4 | 37 | 156 |
| 2011 | 0 | 4 | 1 | 3 | 0 | 2 | 1 | 3 | 3 | 3 | 2 | 3 | 25 | 148 |
| 2012 | 2 | 1 | 3 | 4 | 6 | 2 | 2 | 1 | 2 | 5 | 2 | 2 | 32 | 170 |
| 2013 | 2 | 0 | 2 | 0 | 2 | 2 | 4 | 2 | 1 | 1 | 4 | 3 | 23 | 148 |
| 2014 | 0 | 1 | 4 | 4 | 4 | 6 | 6 | 0 | 3 | 1 | 1 | 5 | 35 | 179 |
| 2015 | 3 | 0 | 1 | 4 | 4 | 1 | 3 | 1 | 6 | 1 | 5 | 1 | 30 | 165 |
| 2016 | 2 | 1 | 6 | 4 | 6 | 1 | 3 | 2 | 6 | 3 | 6 | 1 | 41 | 111 |
| 2017 | 4 | 1 | 2 | 2 | 1 | 4 | 2 | 4 | 2 | 3 | 1 | 1 | 27 | 138 |
| 2018 | 0 | 2 | 0 | 3 | 4 | 4 | 1 | 0 | 0 | 2 | 0 | 0 | 16 | 175 |
| Total | 76 | 84 | 84 | 101 | 110 | 80 | 88 | 81 | 92 | 105 | 93 | 89 | 1083 |  |

**Methods of data analysis**

Metric of birth seasonality

In order to assess birth seasonality, we chose to use the classic metric of birth amplitude [e.g. 12, 35] defined as the *percentage deviation in the number of births from the annual monthly mean* and hereafter written as *Dev.Birth.* This metric has the advantage of de-trending potential year-to-year fluctuations in numbers of births [18, 69]. It was calculated as the difference between the number of births in specific months and years (e.g. number of births in January 1992) and the mean number of births per month over the same year (e.g. average monthly births in 1992), divided by the monthly mean for the year. *Dev.Birth* is month and year specific and is obtained with the following equation:

*Dev.Birth_ij_=N_ij_-M_j_M_j_,*

where *N* is the number of births in a given month (i) of a given year (j) and M the mean number of births per month in a given year (j).

Due to the small sample size, the data show wide variations in the number of observed births across years and months (mean = 2.65, SD=1.78, ranging from 0 to 8). On the one hand, we wanted to keep the variance between years to account for temporal fluctuations in births between years, but on the other hand, the small sample size implies wide fluctuations in the observed number of births for a given month of a given year. To capture potential changes in births patterns across years while decreasing the noise resulting from sampling variance, we median-smoothed the number of births in a given month across three successive years (with the ‘smooth’ function in the ‘stats’ package in ‘R’ (R Core Team, 2020), using the ‘3’ methods and a ‘copy’ end rule). We then calculated the percentage of monthly deviation in relation to the annual mean.

Due to the loss of birth records between 1983 and 1987, some of the data were missing. For descriptive statistics and correlation tests, we merged the data for 1982 with the 1988 data (as if there were no gap within those years) but not for trends analyses across years (where only the years from 1988 to 2018 were kept).

As indicated above, the number of women of child-bearing age is expected to be constant through years. However, because changes in the number of births across years can be due to differences in the number of women present in the village, we further investigated this potential bias. Our results confirmed that the data for the number of births are valid to study birth patterns in this population (S Text, S1 Fig).

Seasonal fluctuation in births

To test for seasonal fluctuations in birth patterns we performed sinusoidal regressions so that:

Where *X_t_* is the percentage deviation in number of births from the annual monthly mean (*Dev. Birth*), *t* is the month of the year, and *f* is the frequency of periodic variation. Sinusoidal models such as these have been previously used to characterize the seasonality of various events [e.g. 4, 70, 71]. More specifically, we tested two wavelengths: 2π/12 and 2π/6 corresponding respectively to a unimodal or bimodal distribution of *Dev.Birth* per year.

Birth seasonality and climatic variables

Data for precipitation and temperature for each month of each year are, respectively, the monthly mean daily precipitation (in mm) and the monthly mean temperature (in Cº) (S1 and S2 Tables).

We use linear regression to investigate whether four explanatory variables – precipitation (daily mean for each month in mm, S1 Table) and temperature (monthly mean in Cº, S2 Table) at birth and conception (taken as 9 months before birth) – explain birth seasonality (Dev.Birth) within the year. More specifically, we tested all potential models combining the four explanatory variables and their interactions together with the mean temperature and precipitation per years using the ‘dredge’ function of the ‘MuMIn’ package in ‘R’ and we ranked them as a function of the AICc [72]. Among the best models (ΔAICc<2), we chose the most parsimonious.

Change across years in the timing of seasonal birth peaks

As an initial investigation of potential change in birth seasonality over time, analyses were performed for the years before 2003, which represent half of our time series, and for the years 2003 and after. As some differences were found between these two samples (Fig 1) an analysis in-depth was performed. To assess whether the birth pattern might have changed over the years, we analysed the potential changes in the months for which *Dev.Birth* was at its minimum and maximum each year from 1988 to 2018. We chose this method because we suspect birth patterns to be bimodal with possibly different changes in the timing of the two peaks across years, which would be difficult to detect using harmonic regression. We indexed the months by values from 1 to 12. For each year, we then looked for the month(s) where *Dev.Birth* was at its minimum between the months of November and February (hereafter written as ‘Minimum 1’) and between May and August (hereafter written as ‘Minimum 2’). These were chosen based on the timing of the troughs in the bimodal distribution revealed by previous analysis (see results below). When there were several equal minimum values for these periods, the mean indexes for the months were used. Finally, we looked for the maximum values in between these two consecutive minimum values, thus locating the two corresponding birth peaks (hereafter written as ‘Peak1’ and ‘Peak 2’) in relation to the birth minimums. To assess changes in the timing of these minimums and maximums, we performed linear regressions of the timing of these yearly troughs and peaks for the years from 1988 to 2018.

**Results**

Birth Seasonality

The *percentage deviation in the number of births in the annual monthly mean* (*Dev.Birth*) before and after year 2003 (the mid-point of the following period) is shown in Fig 1. Although there is more variation across years in years 1980-1982 and 1988-2003 than in years 2003-2018, figs 1A and 1B both exhibit a seasonal distribution of births across the year with two modes. This is further confirmed by sinusoidal regression favouring a wavelength of 2π/6 with p=1.834e-05 (*a*=-4.18 and *b*=-10.75) for the years before 2003 and for 2003 and after, with p=1.462e-05 (*a*=O.65 and *b*=-19.47) for the years after 2003. The month of birth accounts for 8.4% of the variance in *Dev.Birth* (respectively 4.6% before 2003 and 11% in 2003 and after). S2 Fig shows similar results for the monthly, non-smoothed, number of births over the whole period, and S1 Fig shows similar results for monthly fertility rates for the years for which we have a good estimate of women of reproductive age, showing that potential interannual fluctuations in the number of women do not bias our results (Supplementary text).

**Fig 1.** Boxplots showing Dev.Birth median-smoothed across three successive months for the years 1980-1982 and 1989-2003 (A) and 2003-2018 (B) and the corresponding fitted sinusoidal regression shown by the red line. The average peak and trough months are marked and determined as: ‘minimum 1’ (lowest number of births between November and February,▽), ‘maximum 1’ (highest number of births between ‘minimum 1’ and ‘minimum 2’, △), ‘minimum 2’ (lowest number of births between May and August, ▼ ) and ‘maximum 2’ (highest number of births between ‘minimum 2’ and ‘minimum 1’,▲).

The two bimodal distributions show an initial negative deviation in November-February before 2003 and in December-January after 2003. The first wave of positive values is in March-April for the years before 2003 and in April-May for the years after 2003. The second wave of negative values is in June-July for the years before 2003 and in July-August after 2003, followed by a second wave of positive values in August-October for the years before 2003 and in October-November for the years after 2003. Assuming a 9-month pregnancy, these results suggest that the corresponding peaks in conceptions would be in June-July and November-December for the years before 2003 and in July-August and January-February for the years after 2003.

The two peaks occur at an interval of about 6 months (Fig 1). The average 2.5-year IBI for the Baka is equivalent to five 6-month cycles (Ramirez Rozzi 2018). This means that successive sibling births will occur alternately in one peak or the other. Therefore the IBI of 2.5 years in the Baka is linked, as expected, with a birth seasonality pattern with two peaks.

Birth seasonality and climatic variables

Precipitation data from the study site (S1 Fig and S1 Table) show a bimodal pattern through the year with two rainy seasons (March-May and August-November) and two dry seasons (more intense in December-February and less intense in June-August) at 6-monthly intervals. Temperature is moderate to high all year round (daily mean of ~23ºC) with no wide yearly oscillation (S1 Fig and S2 Table). Weather conditions govern the Baka economic calendar (Fig 2): hunting is especially important during rainy seasons whereas fishing and bush mango gathering are intense during dry seasons [65, 66]. Subsistence activities clearly follow climatic fluctuations (Fig 2) so it can be difficult to distinguish whether a specific outcome is influenced by subsistence activities or by the climate.

**Fig 2.** Daily mean precipitation (mm) and temperature (Cº) by month for the years 1979-2002 (dark blue bars and dark red line) and 2003-2018 (light blue bars and light red line), and corresponding timeline of predominant economic activities. It must be stressed that, due to the bimodal distribution of precipitation through the year, precipitation in a given month is strongly inversely correlated with precipitation 9 months later (cor=-32%, p=8.5e-11). Temperature in the 9-month intervals is less correlated (cor=9%, p=0.06). Data from ERA5 [68].

We ran 452 models testing the effects on Dev.Birth of combinations of the four explanatory variables (precipitation and temperature at conception and at birth) and their interactions together with the mean annual temperature and precipitation. Of these models, 12 were ranked as best (ΔAICc<2). Among these, two alternative models are equally parsimonious, incorporating precipitation and temperature either at birth or at conception (presented in Table 2). Using monthly deviations from annual precipitation and temperature means as explanatory variables gives similar results (not shown). Our results demonstrate a significant positive effect of precipitation and temperature at birth and a negative effect of precipitation and temperature at conception (Table 2).

**Table 2. Linear regression between *Dev.Birth* and climatic variables at birth and conception.**

|  | Model 1 | | | Model 2 | | |
| --- | --- | --- | --- | --- | --- | --- |
| Predictors | Estimates | CI | p | Estimates | CI | p |
| (Intercept) | -175.08 | -326.12 – -24.05 | 0.023 | 185.23 | 34.62 – 335.85 | 0.016 |
| prec.birth | 4.26 | 1.56 – 6.97 | 0.002 |  |  |  |
| temp.birth | 6.71 | 0.51 – 12.92 | 0.034 |  |  |  |
| Prec.conc | - | - | - | -3.98 | -6.65 – -1.31 | 0.004 |
| Temp.conc | - | - | - | -7.19 | -13.38 – -0.99 | 0.023 |
| Observations | 408 | | | 408 | | |
| R^2^ / R^2^ adjusted | 0.025 / 0.021 | | | 0.024 / 0.020 | | |

Change across years in the timing of seasonal birth peaks

Detectable changes in rainfall and temperature have been recorded over the last 30 years in the region of Le Bosquet. Weather data from 1979 to 2018 show an overall decrease in precipitation of about 30% and an increase in temperature of about 1.15ºC (Fig 3). These changes have affected the magnitude of these weather variables but have not modified their pattern of occurrence over the year, which is observed to be the same before and after year 2003 (Fig 2).

**Fig 3.**Total amount of yearly rainfall (mm) and daily mean temperature (ºC) for years 1979-2018. The regression lines highlight the decrease in rainfall (p=2.13e-6) and the increase in temperature (p=1.16e-8). Data from ERA5 [68]

As explained in the Fig 1 legend, ‘minimum 1’ is the lowest number of births between November and February, while ‘minimum 2’ is the lowest number of births between May and August. Then there is ‘maximum 1’ which is the highest number of births between ‘minimum 1’ and ‘minimum 2’ and also ‘maximum 2’ as the highest number of births between ‘minimum 2’ and ‘minimum 1’. Linear regression on the timing of ‘minimum 1’ and ‘maximum 1’ show no changes in their timing over the years (p>0.1, results not shown). However, in the last 30 years, the timing of ‘minimum 2’  and ‘maximum 2’ has shifted significantly to later in the year (p<0.01, Fig 4), roughly from late May to mid-July and from late August to late October respectively. Applying a similar method to precipitation and temperature data confirmed that, conversely, there were no significant changes in the timing of precipitation and temperature over the years. But our results show a decline and an increase, respectively, in the magnitudes of precipitation and temperature from 1979 to 2018 (but with no change in their timing, see Fig 3 and S1 Table). These changes occur in parallel with a change in the timing (but not in the magnitude) of births in the second part of the year.

**Fig 4.** Months when *Dev.Birth* is at a minimum between May and August (i.e., ‘minimum 2’, ▼) and at a maximum between the two minimums (i.e., ‘maximum 2’, ▲). The linear regression lines have coefficients of 0.06048 (p-value=1.49e-5) for ‘minimum 2’ and 0.0764 (p-value=0.000321) for ‘maximum 2’. The grey areas show the 099 confidence intervals of mean predictions.

**Discussion**

This study brought several findings: 1) the presence of birth seasonality among the Baka; 2) the relationship between birth seasonality with meteorological variables, and 3) local evidence of climate change in south-eastern Cameroon and its relationship with the Baka birth pattern. We discuss these findings with regard to the socio-economic activities of the Baka.

Our analyses demonstrate significant two-peak seasonality in births among the Baka. This pattern correlates well with rainfall fluctuation over the year. Multivariate analysis demonstrates that precipitation at conception or at birth potentially have effects, respectively negative and positive, on the monthly number of births. Note that because we do not have any information on the number of conceptions that do not result in births, we cannot perform two sets of analyses for, respectively, the number of conceptions and births per month. We were not able to distinguish whether these are independent effects or whether they arise because of the co-linearity with the climatic variables. In both cases, however, there is a combined mechanism at work between temperature and rainfall, as found by others [3]. In this sense, temperature might not correlate directly with conceptions and births (via biological or behavioural mechanisms) but it might have a role in controlling other variables that do affect the birth pattern. Together, the climatic variables accounted for 30% of the variance in *Dev.Birth* explained by the month of birth.

Health, nutrition and social issues related to specific seasons could provide us with potential explanations for the occurrence of these peaks in conception in the dry seasons (Fig 2). Conception outcomes are better in dry periods because the incidence of infectious diseases is lower than during rainy periods in tropical regions [28, 30, 36]. Berry et al. [73] have shown that in regions where malaria is endemic, the prevalence of malaria is greater in women who become pregnant during the rainy season than during the dry season. Specific data on the incidence of malaria, or any other infectious disease, do not exist for the Baka. However, Cameroon has a relatively high prevalence of malaria [74] and the Baka are affected by this disease, as suggested by the number of plants and remedies they know for treating it [75], so we cannot omit this possible determinant.

A good nutritional status has been pointed out as a key factor for higher conception rates [12, 33, 36]. Although our study has not collected actual data on energy intake, our knowledge on time allocation, dietary diversity and resource availability among the Baka does allow speculation as to the energy available over the year [76-78]. While there is no record of severe starvation among the Baka [44], dry seasons are likely to ensure a better nutritional status than the rainy seasons, when the Baka diet is less varied with less frequent consumption of starchy staples, meat, fruits and vegetables [77]. Specifically during the dry seasons, the overall availability of wild food is greater [i.e. 44, 45]. For instance, wild yams (*Dioscorea sp.*), one of the main wild sources of carbohydrates and calories for the Baka [45], are larger and consumed more frequently during dry seasons, while in rainy seasons, yam tubers are small and only consumed in cases of necessity [45]. The yam preferred by the Baka, *D. praehensilis,* is most available in the wild in the month of December [76]. More specifically, the less intense dry season, in which we found the August conception peak, is characterized by a higher intake of bush mango [65], which can provide up to 50% of the calorie intake [46] thanks to the high proportion of lipids in the fruit [79]. Wild meat and fish are also more frequently consumed during the more intense dry season, which matches the first conception peak in January [80]. The inclusion of fish, wild meat and larger amounts of starchy yam might improve the nutritional status of the population during this period. Conception peaks in the Baka thus seem to be concentrated in periods of better nutrition. These insights are consistent with two previous studies which found that conception patterns are related to better nutritional status among hunter-gatherers [37, 38] and additional studies on agricultural societies [12, 13, 29].

Other socio-cultural factors could play a role in birth seasonality in the Baka, such as mobility patterns and subsistence and social activities, which are directly relevant to physical and emotional proximity among the Baka and may thus affect the frequency of intercourse in the population [81]. The fact that conception rates are low during rainy seasons is not surprising if we consider the mobility pattern of the Baka. In these periods, men are more frequently involved in hunting expeditions (especially during the more intense rainy season, from September to November). While they are away, the women are symbolically involved in the success of the men's hunting and have to respect socio-cultural practices and norms in order to avoid hunting failure, including avoidance of extramarital relations [48]. During the longer rainy season, the Baka are also more engaged in harvesting cacao beans for their Bantu neighbours [82]. During this period, the Bantu hire the Baka, mostly men, for several days to several weeks, taking them to their villages. The women and children sometimes join their men in the Bantu villages but sometimes stay home, which therefore also affects the couple's interactions. In contrast, during the dry seasons, families are more likely to be together. This proximity could be even greater during the more intense dry season, as Baka households tend to live in their forest camps in small numbers. The small size of these camps (30-50 individuals), made up of close relatives, might give the couples more intimacy. As privacy could favour more frequent sexual intercourse [83], more conceptions would be likely during this period. In August, however, the Baka tend to be more present in the village, mostly to dig new farm plots and carry out agricultural tasks [84]. Gathering expeditions to the forest and forest camps take place, but much less frequently than in the other dry season.  Therefore, in the Baka lifestyle, mobility patterns related to subsistence and social activities could play a role in birth seasonality.

Other studies have shown changes in the magnitude [47, 81] or the shape of the birth pattern over time [11]. Our study also shows such a change: a decrease/increase in precipitation/temperature within the last 30 years parallel to a shift in the timing of the second peak of births in the year. Although we do not demonstrate causality between these two effects, we can hypothesize that a change in food availability due to the decline in precipitation may affect the dynamics of subsistence activities or of the energy balance, which in turn has an impact on the timing of births. For instance, the decline in precipitation through the entire year may have a greater effect on food availability during the more intense dry season - producing a clear impact on births at the second peak occurring 9 months later – and a lesser effect in the less intense dry season (June and July) producing a negligible effect on the first peak. Whatever the reason, these results appear to suggest that the impact of climate change has already started to affect hunter-gatherer life patterns.

In conclusion, this study has shown that there is a clearly seasonal pattern of births among the Baka with two main peaks each year, that conceptions mainly occur during the dry seasons and, finally, that a shift in the peak of births seems to relate to climatic changes over the years.

**Bibliography**

1. Rau R. Seasonality in Human Mortality. Springer; 2006.
2. Karlsson L. Indigenous infant mortality by age and season of birth, 1800–1899: Did season of birth affect children’s chances for survival?. Int J Environ Res Public Health. 2018;15(1):1-12
3. Boland MR, Fieder M, John LH, Rijnbeek PR, Huber S. Female reproductive performance and maternal birth month: a comprehensive meta-analysis exploring multiple seasonal mechanisms. Sci Rep. 2020;10:555.
4. Douros K, Fytanidis G, Papadimitriou A. Effect of the month of birth on the height of young adult males. Am J Phys Anthropol. 2019;170(3): 1–4.
5. Doblhammer G, Vaupel JW. Lifespan depends on month of birth. Proc Natl Acad Sci U S A. 2001:98(5):2934–2939.
6. Gagnon A. Effect of birth season on longevity: thrifty and hopeful phenotypes in historical Quebec. Am J Hum Bio. 2021; 24(5):654–660.
7. Wang C, Zhang Y. Season of birth and schizophrenia: Evidence from China. Psychiatry Res. 2017;253:189–196.
8. Schnittker J. Season of birth and depression in adulthood: Revisiting historical forerunner evidence for in-utero effects. SSM Popul Healt.2018; 4:307–316.
9. Zhang Y, Devore EE, Strohmaier S, Grodstein F, Schernhammer ES. Birth month, birth season, and overall and cardiovascular disease mortality in US women: Prospective cohort study. The BMJ. 2019;367:1–10.
10. Cowgill UM. The Season of Birth in Man. Man. 1966; 1(2): 232–240.
11. Cypryjański J. Changes in seasonality of births in Poland in the years 1900-2009. Demogr Res. 2019;40:1441–1454.
12. Dorélien AM. Birth seasonality in Sub-Saharan Africa. Demogr Res. 2016;34(1):761–796.
13. Bailey RC, Jenije MR, Ellison PT, Bentley GR, Harrigan AM, Peacock NR. The ecology of birth seasonality among agriculturalists in central Africa. Journal of Biosocial Science. 1992;24(3):393–412.
14. Dubost G, Henry O. Seasonal reproduction in neotropical rainforest. Zoological studies. 2017;56(2):1-9.
15. McNutt JW, Groom R, Woodroffe R. Ambient temperature provides an adaptive explanation for seasonal reproduction in a tropical mammal. Journal of Zoology. 2019;309:153-160.
16. Boesch C, Boesch-Achermann H. The Chimpanzees of the Taï-Forest: Behavioural Ecology and Evolution. Oxford university press; 2000.
17. Hsu MJ, Agoramoorthy G, Lin J. Birth seasonality and interbirth intervals in free-ranging Formosan Macaques, *Macaca cyclopis*, at Mt. Longevity, Taiwan. Primates. 2001;42(1):15-21.
18. Heldstab SA, van Schaik CP, Müller DWH, Rensch E, Lackey LB, Zerbe P, et al. Reproductive seasonality in primates: patterns, concepts and unsolved questions. Biological reviews. 2020; 000-000.
19. Kiltie RA. Gestation as a constraint on the evolution of seasonal breeding in mammals in Evolution of Life Histories of Mammals. Theory and Pattern (ed. Boyce, M. S.) 257–289. Yale University Press, New Haven; 1988.
20. Ellison PT, Valeggia CR, Sherry DS. Human birth seasonality in Seasonality in Primates: Studies of Living and Extinct Human and Non-Human Primates (ed. Brockman, D. K., Van Schaik, C. P.). Cambridge University Press; 2005.
21. Knott CD. Energetic responses to food availability in the great apes: implications for hominin evolution. In Seasonality in Primates: Studies of Living and Extinct Human and Non-human Primates (ed. Brockman, D. K., Van Schaik, C. P.) 351–378. Cambridge University Press, Cambridge; 2005.
22. Ferguson G. Some aspects of birth seasonality in Kenya. Soc Sci Med. 1987;25(7):793–801.
23. Grech V, Savona-Ventura C, Agius-Muscat H, Janulova L. Seasonality of births is associated with seasonality of marriages in Malta. Journal of Biosocial Science. 2003;35:95-105.
24. Herteliu C, Ileanu BV, Ausloos M, Rotundo G. Effect of religious rules on time of conception in Romania from 1905 to 2001. Human Reproduction. 2015;30(9):2202–2214.
25. Condon RG, Scaglion R. The ecology of human birth seasonality. Human Ecology. 1982;10(4):495-511.
26. Lam DA, Miron JJA. The effects of temperature on human fertility. Demography. 1996;33(3):291-305.
27. Ruiu G, Breschi M. Seasonality of livebirths and climatic factors in Italian regions (1863-1933). Historical life course studies. 2017;4:145-164.
28. Bantje H. Seasonality of births and birthweights in Tanzania. Soc Sci Med. 1987;24(9):733–739.
29. Leslie PW, Fry PH. Extreme seasonality of births among nomadic Turkana pastoralists. Am J Phys Anthropol. 1989;79(1):103–115.
30. Philibert A, Tourigny C, Coulibaly A, Fournier P. Birth seasonality as a response to a changing rural environment (Kayes region, Mali). Journal of Biosocial Science. 2013;45(4):547–565.
31. Levine RJ. Male Factors Contributing to the Seasonality of Human Reproduction. Ann N Y Acad Sci. 1994;709(1):29–45.
32. Levine RJ, Brown MH, Bordson BL, Stanley JM, Mathew RM, Starr TB. Deterioration of semen quality during summer in New Orleans. Fertil Steril. 1988;49(5):900–907.
33. Ruiu G, Breschi M. Intensity of Agricultural Workload and the Seasonality of Births in Italy. European Journal of Population. 2020;36(1):141–169.
34. Buckles KS, Hungerman DM. Season of birth and later outcomes: old questions, new answers. The review of economics and statistics. 2013;95(3):711-724.
35. Martinez-Bakker M, Bakker KM, King AA, Rohani P. Human birth seasonality: latitudinal gradient and interplay with childhood disease dynamics. Proc. R. Soc. B. 2014;281.
36. MacVicar S, Berrang-Ford L, Harper S, Steele V, Lwasa S, Bambaiha, DN, Twesigomwe S, et al. How seasonality and weather affect perinatal health: Comparing the experiences of indigenous and non-indigenous mothers in Kanungu District, Uganda. Soc Sci Med. 2017;187:39-48.
37. Wilmsen EM. Seasonal effects of dietary intake on Kalahari San. Fed Proc. 1978;37(1):65–72.
38. Hill K, Hurtado AM. Aché Life History: the ecology and demography of a foraging people. Aldine de Gruyter; 1996.
39. Wilmsen EN. Studies in diet, nutrition and fertility among a group of Kalahari Bushmen in Botswana. Social Science lnformation. 1982;21(1):95-125.
40. Pachauri RK, Meyer L. et al. Climate change 2014: synthesis report. Contribution of Working Groups I, II and III to the fifth assessment report of the Intergovernmental Panel on Climate Change. IPCC; 2014.
41. International labour Organization, 2017. Indigenous peoples and climate change: From victims to change agents through decent work (Geneva, Switzerland).
42. Ford JD. Indigenous health and climate change. American journal of public health. 2012;102(7):1260-1266.
43. Ramirez Rozzi FV, Koudou Y, Froment A, Le Bouc Y, Botton J. Growth pattern from birth to adulthood in African pygmies of known age. Nature Communications. 2015;6:1–12.
44. Ramirez Rozzi FV. Reproduction in the Baka pygmies and drop in their fertility with the arrival of alcohol. Proc Natl Acad Sci U S A. 2018;115(27):6126-6134.
45. Dounias E. The management of Wild Yam tubers by the Baka Pygmies in Southern Cameroon. African Study Monographs Supplementary Issue. 2001;26:135-156.
46. Yasuoka H. Fledging agriculturalists? Rethinking the adoption of cultivation by the Baka hunter-gatherers. African Study Monographs Supplementary Issue. 2012;43:85–114.
47. Khajavi A, Pishgar F, Parsaeian M, Moghaddam SS, Jeddian A, Bahrami-Taghanaki HR, et al. Birth seasonality in rural areas of Iran, analysis of 5,536,262 births from 1992 to 2007. Annals of Epidemiology. 2016;26(12):846-852.
48. Duda R. Ethnoecology of hunting in an empty forest. Practices, local perceptions and social change among the Baka (Cameroon). Unpublished doctoral thesis. Universitat Autònoma de Barcelona. Barcelona, Spain; 2017.
49. Verdu P, Austerlitz F, Estoup A, Vitalis R, Georges M, Théry S, et al. Origins and Genetic Diversity of Pygmy Hunter-Gatherers from Western Central Africa. Current Biology. 2009;19(4):312–318.
50. Cavalli-Sforza LL. African Pygmies. Academic Press; 1986.
51. Becker NSA, Verdu P, Froment A, Le Bomin S, Pagezy H, Bahuchet S, et al. Indirect evidence for the genetic determination of short stature in African pygmies. Am J Phys Anthropol. 2011;145:390–401.
52. Becker NSA, Verdu P, Georges M, Duquesnoy P, Froment A, Amselem S, et al. The role of GHR and IGF1 genes in the genetic determination of African pygmies’ short stature. Eur J Hum Genet. 2013;21:1–6.
53. Pemberton TJ, Verdu P, Becker NS, Willer CJ, Hewlett BS, Le Bomin S, et al. A genome scan for genes underlying adult body size differences between Central African hunter-gatherers and farmers. Hum Genet. 2018;137:487-509.
54. Perry GH, Foll M, Grenier JC, Patin E, Nédélec Y, Pacis A, et al. Adaptive, convergent origins of the pygmy phenotype in African rainforest hunter-gatherers. Proc Natl Acad Sci U S A. 2014;111:3596–3603.
55. Verdu P, Leblois R, Froment A, Théry S, Bahuchet S, Rousset F, et al. Limited dispersal in mobile hunter − gatherer Baka Pygmies. Biol Lett. 2010;6:858–861.
56. Maley J, Brenac P. Vegetation dynamics, paleoenvironments and climatic changes in the forests of western Cameroon during the las 28,000 years B. P. Review of Palaeobotany and Palynology. 1998;99:157-187.
57. Vincens A, Schwartz D, Elenga H, Reynaud-Farrera I, Alexandre A, Bertaux J, et al. Forest response to climate changes in Atlantic Equatorial Africa during the last 4000 years BP and inheritance on the modern landscapes. J Biogeog. 1999; 26:879-885.
58. Plana V. Mechanisms and tempo of evolution in the African Guineo-Congolian rainforest. Phil Trans R Soc Lond B. 2004;359:1585-1594.
59. Molua EL. Climatic trends in Cameroon: implications for agricultural management. Climate research. 2006;30:255-262.
60. Hayashi K. Hunting activities in forest camps among the Baka Hunter-Gatherers of southeastern Cameroon. African Study Monographs. 2008;29(2):73-92.
61. Molua EL. Climate variability, vulnerability and effectiveness of farm-level adaptation options: the challenges and implications for food security in Southwestern Cameroon. Environmental and Development Economics. 2002;3:529-545.
62. Yamauchi T, Sato H, Kawamura K. Nutritional status, activity pattern, and dietary intake among the Baka Hunter-Gatherers in the village camps in Cameroon. African Study Monographs. 200;21(2):67-82.
63. Yasuoka H. The sustainability of Dukier (Chepalophus Spp.) hunting for the Baka Hunter-Gatherers in southeastern Cameroon. African Study Monographs Supplementary Issue. 2006;33:95-120.
64. Kitanishi K. Seasonal changes in the subsistence activities and food intake of the Aka hunter-gatherers in northeastern Congo. African Study Monographs. 1995;16:73-118.
65. Gallois S, van Andel T, Heger T, Sonké B, Henry AG. Comparing apples and pears: the hidden diversity of the Central African Bush Mangoes (Irvingiaceae). Economic Botany, in press. 2020
66. Gallois S, Duda R. Beyond productivity: The socio-cultural role of fishing among the Baka of southeastern Cameroon. Revue d’ethnoécologie. 2016;10:0–27.
67. Reyes-García V, Díaz-Reviriego I, Duda R, Fernández-Llamazares Á, Gallois S. Hunting Otherwise. Human Nature. 2020;31(3):203-221.
68. Copernicus Climate Change Service (C3S) (2017): ERA5: Fifth generation of ECMWF atmospheric reanalyses of the global climate. Copernicus Climate Change Service Climate Data Store (CDS), date of access 20/11/2019. [https://cds.climate.copernicus.eu/cdsapp#!/home](https://cds.climate.copernicus.eu/cdsapp#!/home)
69. He D, Earn D. Epidemiological effects of seasonal oscillations in birth rates. Theoretical Population Biology. 2007;72(2):274–291.
70. Jensen CB, Gamborg M, Raymond K, McGrath I, Sørensen TIA. Secular trends in seasonal variation in birth weight. Early human development. 2015;91:361-365.
71. Fellman J. Demographic Seasonality. Twin Research and Human Genetics. 2019;22(3):187–194.
72. Burnham KP, Anderson DR. Model Selection and Multimodel Inference: A Practical Information-theoretic Approach. Springer-Verlag; 2002.
73. Berry I, Walker P, Tagbor H, Bojang K, Coulibaly SO, Kayentao K, et al. Seasonal dynamics of malaria in pregnancy in west Africa : evidence for carriage of infections acquired before pregnancy until first contact with antenatal care. American Journal of Tropical Medicine and Hygiene. 2018;98(2):534–542.
74. Antonio-Nkondjio C, Ndo C, Njiokou F, Bigoga JD, Awono-Ambene P, Etang J, et al. Review of malaria situation in Cameroon : technical viewpoint on challenges and prospects for disease elimination. Parasites and Vectors. 2019;12:501.
75. Betti JL. An ethnobotanical Study of medicinal plants among the Baka pymies in the Dja biopher reserves, Cameroon. African Study Monographs. 2004;25(1):1–27.
76. Gallois S, Heger T, van Andel T, Sonké B, Henry AG. From Bush Mangoes to Bouillon Cubes: Wild Plants and Diet among the Baka, Forager-Horticulturalists from Southeast Cameroon. Economic Botany. 2020:1–13.
77. Gallois S. Growing up in a changing world. A case study among Baka children (southeastern Cameroon). AnthropoChildren. 2017;7:1-20.
78. Reyes-Garcia V, Powell B, Díaz-Reviriego I, Fernández-Llamazares Á, Gallois S, Gueze M. Dietary transitions among three contemporary hunter-gatherers across the tropics. Food security. 2019;11:109-122.
79. Terashima H, Ichikawa M. A comparative ethnobotany of the Mbuti and Efe hunter-gatherers in the Ituri forest, Democratic Republic of Congo. African Study Monographs. 2003;24:1-168.
80. Duda R, Gallois S, Reyes-García V. Ethnozoology of bushmeat. Importance of wildlife in diet, food avoidances and perception of health among the Baka (Cameroon). Revue d’ethnoécologie. 2018;14.
81. Lam DA, Miron JJA. Seasonality of births in human populations. Biodemography and Soc Biol. 1991;38(1–2):51–78.
82. Oishi T. Cash crop cultivation and interethnic relations of the Baka hunter-gatherers in southeastern Cameroon. African Study Monographs Supplementary Issue. 2012;43:15-136.
83. Diamond J. Why is Sex Fun? Basic Books; 1997.
84. Leclerc C. L’adaptation de l’agriculture chez les Pygmées baka du Camerou. Editions Quae; 2012

**Acknowledgements**

Thanks to X. Garde, B. Bordage and to all the staff of IRD Yaounde for their logistical support; to the nuns at the Le Bosquet mission for their kind hospitality and especially to P. Kalo, J. B. Etoa and other Baka collaborators for their help, assistance and friendship. We also want to thank A. Epelboin for his useful comments on the previous manuscript; Elisa Bergas Massó, who provided us with high quality meteorological data without which the study could not be carried out, and Mikel Larrañaga Arcay. This study was supported by the CNRS and IRD, and by grants from the Wenner–Gren Foundation (7810) (FRR), the National Geographic (8863-10) (FRR) and the Agence Nationale de la Recherche under the Blanc SVSE7-2011 GrowinAP programme (FRR).

**Author’s contribution**

LPF, FRR: obtain data

LPF, SP, FRR: Designed research, performed research

LPF, SG, SP, FRR: analyzed data, wrote the paper.

**Competing interests**

The authors declare no competing interests
