# Supplementary material for: Reproductive seasonality in the Baka Pygmies, environmental factors and climatic changes
Source: PLoS One. 2022 Mar 8;17(3):e0264761. doi: 10.1371/journal.pone.0264761 (PMC8903253; doi:10.1371/journal.pone.0264761)
Supplement: Attachment — Submitted filename: Response-to-reviewerst.docx [file pone.0264761.s011.docx]

**Response to reviewer**

Thank you to the reviewer and the Academic Editor for their comments.

There is no change in the financial disclosure.

Laboratory protocols are not applicable to this study.

ACADEMIC EDITOR: This is an well done study that now meets Plos One criteria for publication. Please consider my decision of minor revision as an opportunity to finalize any small editorial changes. We do not have copy editing services, and I noticed a couple typos.

We have realized the editorial (typo) changes (lines 13, 14, 28, 37, 42, 45, 68, 78, 79, etc.)

Journal Requirements:

References

References were checked and no change was needed.

Reviewers' comments:

The reviewer suggested some minor corrections, the following

Minor corrections:

line 100: "The rainy seasons are characterized by short periods of torrential rainfall"

line 156: "Subsistence activities vary according to the seasons, ..."

line 194: "This study has obtained approval from..."

line 360: "This study brought several findings: ..." is awkward. Maybe "This study had several results: ..." or similar.

Of course, these suggestions were all accepted and can be seen in the maked-up-copy of the ms.

The figures are now PACE generated ones.
